# Extreme Droughts Push Heterotrophic Functions Above Baseline Levels in a Neotropical Ecosystem

**DOI:** 10.1111/gcb.70777

**Published:** 2026-03-03

**Authors:** Thibaut Rota, Vincent E. J. Jassey, Céline Leroy, Jean‐François Carrias, Bruno Corbara, Joséphine Leflaive, Arthur Compin, Diane S. Srivastava, Vinicius F. Farjalla, Régis Céréghino

**Affiliations:** ^1^ Centre de Recherche Sur la Biodiversité et L'environnement Université de Toulouse, CNRS, IRD Toulouse France; ^2^ Institute of Microbiology University of Applied Sciences and Arts of Southern Switzerland Mendrisio Switzerland; ^3^ Forest Entomology Swiss Federal Research Institute WSL Birmensdorf Switzerland; ^4^ Department of Ecology, Faculty of Environmental Sciences Czech University of Life Sciences Prague Praha 6–Suchdol Czech Republic; ^5^ AMAP Univ. Montpellier, CIRAD, CNRS, INRAE, IRD France; ^6^ EcoFoG, AgroParisTech CIRAD, CNRS, INRAE, Université Des Antilles, Université de Guyane, Campus Agronomique Kourou France; ^7^ LMGE Université Clermont Auvergne, CNRS Clermont‐Ferrand France; ^8^ Department of Zoology & Biodiversity Research Centre University of British Columbia Vancouver Canada; ^9^ Departamento de Ecologia, Instituto de Biologia Universidade Federal do Rio de Janeiro (UFRJ) Rio de Janeiro Brazil

**Keywords:** carbon cycling, climate change, drought intensification, ecosystem multifunctionality, meta‐ecosystems, resilience, resistance

## Abstract

Droughts are intensifying in the humid Neotropics, raising concerns about the impacts on ecosystem processes related to C cycling, such as decomposition and CO_2_ respiration. In particular, the resilience of multiple functions to extreme droughts in Neotropical aquatic systems remains poorly understood, limiting our ability to predict drought‐driven feedbacks on C cycling. Here, we used rain shelters placed above tank bromeliads, plants that hold small freshwater ecosystems within their leaf axils, to emulate drought events ranging from the current norm to different IPCC scenarios. We then quantified the resilience of three key ecosystem functions (microbial respiration, litter decomposition, and photosynthetic efficiency) during a post‐drought rewetting phase of 60 days. To assess the role of biotic recolonization during rewetting, we used mosquito nets over half of the bromeliads to prevent macroinvertebrates from recolonizing bromeliads from adjacent source patches. We found that extreme droughts (94 days) pushed heterotrophic functions above baseline levels during the rewetting phase. Microbial respiration and litter decomposition increased during this rewetting period, relative to undisturbed bromeliads. This boost was even faster when macroinvertebrate recolonization was allowed. Structural equation models suggested that nutrient release from dead organic matter during the rewetting phase, along with changes in bacterial density and shredder biomass, drove the positive shifts in heterotrophic functions and ecosystem multifunctionality. Extreme droughts accelerated C processing in tank bromeliads, particularly when external recolonization occurred, releasing a noticeable amount of carbon to the atmosphere. Our study shed light on the mechanisms underlying post‐drought ecosystem multifunctionality trajectory and its link with C cycling, encouraging future works considering these small but abundant water bodies as sources of C in the Neotropics in the face of drought intensification.

## Graphical abstract

Using tank bromeliads, we experimentally assessed post‐drought resistance and recovery of ecosystem multifunctionality under realistic drought scenarios. We focused on three key functions in these small yet abundant Neotropical freshwater ecosystems: litter decomposition, microbial respiration, and photosynthetic efficiency. We examined mechanisms driving resistance and recovery, including macroinvertebrate recolonization and trophic interactions among bacteria and diverse macroinvertebrate feeding groups. Following an extreme 94‐day drought, we observed a sharp surge in litter decomposition and microbial respiration during rewetting (up to 60 days post‐drought). We discuss the mechanisms behind this pulse of heterotrophic functions above baseline levels after extreme droughts and their implications for carbon cycling in an increasingly dry Neotropical region.

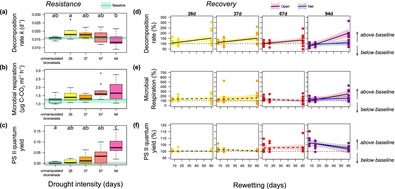

## 1 Introduction

Severe El Niño Southern Oscillation (ENSO) events associated with prolonged dry seasons have been recorded since the mid‐1970's in Neotropical regions (Santer et al. 2005). The Intergovernmental Panel on Climate Change (IPCC) further forecasts severe ENSO events, with a 10%–50% decline in rainfall in the Neotropics over the next decades (Masson‐Delmotte et al. 2021). The increasing frequency and intensity of drought events in the Neotropics are altering the distribution and activity of plants and animals (Dejean et al. 2011). Drought intensification may decrease the capacity of Amazonian forests to store carbon (C) through photosynthesis, raising the concern that they could shift from a net C sink to a net C source in the next decades (Phillips et al. 2009; Gatti et al. 2014; Doughty et al. 2015; Yao et al. 2023). However, the consequences of drought intensification for C‐related ecosystem functioning are less understood in Neotropical freshwater systems.

Understanding the mechanisms influencing ecosystem resilience is key to improve management and suggest mitigation in the face of a changing climate (Oliver et al. 2015). Ecosystem resilience has many definitions and metrics (Holling 1973; Gunderson 2000). Here, we define resilience as the ability of a system to (i) withstand disturbance without changing its inherent functioning (i.e., resistance), and/or (ii) recover to a pre‐disturbance state after the disturbance (i.e., recovery; Ingrisch and Bahn 2018). Resilience to drought is better documented in aquatic systems that periodically dry out, such as ephemeral Mediterranean streams, as their species evolved desiccation‐resistance and dispersal traits allowing the recovery of ecological functions (Boersma et al. 2014). However, aquatic systems in the moist Neotropics were historically less frequently subject to extreme droughts than aquatic systems in more arid regions. Only a handful of studies have examined how drought affects ecosystem functioning in Neotropical aquatic ecosystems (Gutiérrez‐Fonseca et al. 2020; Srivastava et al. 2020; Ruiz et al. 2022; Séguigne et al. 2024a, 2024b), and most of these focused on a single ecosystem function approach (e.g., Pires et al. 2018; Srivastava et al. 2020). As a result, in Neotropical aquatic ecosystems, little is known about the mechanisms driving the resilience following drought of multiple ecosystem functions—coined ecosystem multifunctionality (Pasari et al. 2013; Byrnes et al. 2014). Insurance mechanisms, such as when individual functions respond with different directions to disturbances (Srivastava et al. 2020), or when their responses are lagged in time, may stabilize ecosystem multifunctionality in the face of disturbances (Eisenhauer et al. 2024). Alternatively, consistently positive or negative covariation among the ecological drivers underlying individual functions can result in disproportionate multifunctional alterations.

The multifunctionality of freshwaters in the Neotropics could be severely altered under future drier climates, as organisms in these regions have evolved narrow physiological tolerances due to buffered historical climatic conditions (Tewksbury et al. 2008; Masson‐Delmotte et al. 2021). In aquatic systems occasionally experiencing droughts, we can assume that the current resistance abilities of organisms will enable functional persistence when drought duration falls within historic norms (Céréghino et al. 2020). However, extreme drought events will likely dry out the systems, causing the death of macroorganisms and probably a resetting of functions (Trzcinski et al. 2016). In a meta‐ecosystem context, the recovery of the full set of functions after extreme droughts would require the recovery of functionally‐key macroinvertebrates, either by recolonization from undisturbed, adjacent patches, or from in situ resistance (i.e., from tolerant larvae, and/or from eggs in the dormant “seed bank”; Wisnoski et al. 2019; Bonhomme et al. 2021). Post‐drought trajectories of ecosystem multifunctionality often rely on transient, delayed biological activity, such as nutrient release from dead organisms inducing a lagged boost of microbial respiration and organic matter processing following droughts, as already observed in soils and freshwaters (Göransson et al. 2013; Datry et al. 2018; Kosten et al. 2018). Also key to recovery are patch dynamics, determining extinction‐recolonization dynamics through space and time (Thrush et al. 2009). Most studies of drought resilience in aquatic systems have studied natural drought events (i.e., before‐after control‐impact), or spatial drought gradients (i.e., substituting space for time), particularly in drying river networks (Datry et al. 2014, 2017, 2018; Gutiérrez‐Fonseca et al. 2020). These observational approaches are insightful as they integrate meta‐ecosystem realism (Massol et al. 2017), but they fall short in testing climatic forecast scenarios. To complement existing knowledge, we need replicated experiments emulating drought intensities based on climate scenarios, coupled with experimental control of post‐drought recolonization by organisms, both aspects being highly relevant to the study of multifunctionality resilience in meta‐ecosystems (Massol et al. 2017).

A promising system to address this issue is the aquatic ecosystem within tank bromeliads (Bromeliaceae family, with 3792 species native to the Neotropics; Gouda and Butcher 2024). Tank bromeliads are terrestrial plants holding rainwater and allochthonous detritus within their interlocking leaf axils. These small rain‐fed freshwaters are widespread in Neotropical rainforests, where they form a suitable habitat for a diverse biota, ranging from prokaryotes to macroinvertebrates (Brouard et al. 2012). Despite their small size, bromeliads can be very abundant in Neotropical rainforests, storing large amounts of water (up to 50,000 L ha^−1^; Goffredi, Kantor, and Woodside 2011), facilitating organic matter processing (Leroy et al. 2017), and contributing significantly to C emissions in the Neotropical region (Martinson et al. 2010; Goffredi, Jang, et al. 2011). They contribute disproportionately to the secondary production of macroinvertebrates in forest ecosystems (Dézerald et al. 2018) and fulfil a large array of functions and services (reviewed in Ladino et al. 2019). Owing to their small size and large abundance, they are ideal natural microcosms to test basic questions in ecology (Srivastava et al. 2004) and offer great promise to assess the effects of drought intensification on multifunctional resilience (Srivastava et al. 2020).

Here, we experimentally tested the multifunctional resilience of tank bromeliad ecosystems to IPCC drought duration scenarios (see Figure S1a,b for details on the study design), and how the recolonization by macroinvertebrates after the drought spell may modulate this resilience. We focused on key functions related to C‐cycling: litter decomposition, photosynthetic efficiency, and microbial respiration. Our study addressed three questions. First, which functions would show resistance to drought and thus dominate post‐drought multifunctionality immediately after the disturbance? Second, does the multifunctional recovery following moderate to extreme droughts depend on macroinvertebrate recolonization? Third, how do different types of organisms (i.e., bacteria, fungi, macroinvertebrates) contribute to multifunctional resilience?

Assuming that macroinvertebrates are generally more sensitive to desiccation than unicellular organisms (Vilmi et al. 2020), we hypothesized that functions directly driven by microorganisms, such as photosynthetic efficiency and microbial respiration, would dominate ecosystem multifunctionality after moderate to extreme drought events. Functions driven primarily by macroinvertebrates, such as litter decomposition (Leroy et al. 2017; Ruiz et al. 2022), might recover more slowly, especially if recolonization of shredder invertebrates from external source patches is required to ensure full multifunctional recovery. However, this scenario could be more nuanced. First, microbial communities can alter leaf‐litter decomposition through direct breakdown and priming effects (Danger et al. 2013). Since microorganisms are usually more resilient to drought than macroorganisms (Farjalla et al. 2012), we hypothesized that microbial activity could compensate for the loss of macroinvertebrates after the drought spell, thereby maintaining litter decomposition (Séguigne et al. 2024b). Second, in addition to the leaf shredders, macroinvertebrates in tank bromeliads belong to diverse functional feeding groups (FFGs). Each of these groups is likely to indirectly alter functions primarily driven by microorganisms in different ways. For instance, drought‐induced declines of scrapers and filter‐feeders could reduce their consumption pressure on leaf‐surface biofilms and pelagic microorganisms, respectively, potentially enhancing post‐drought photosynthesis and respiration. Predators may be more sensitive to drought than their prey (e.g., Diptera) (Céréghino et al. 2020), as they may have weaker resistance, dormancy, and recovery capacities compared to their prey (Bonhomme et al. 2021). We suggest that the longer life‐cycles and lower abundances of predators could further delay their recovery after severe droughts compared to their prey (Ruiz et al. 2022), increasing ecosystem multifunctionality through the diminishment of trophic cascades during ecosystem recovery. Finally, under extreme drought scenarios, we hypothesized that heterotrophic functions such as litter decomposition and microbial respiration would increase above baseline levels during rewetting. Bacterial activity is known to increase drastically during rewetting phases following harsh drought events in soils, rivers, lakes, and reservoirs, accelerating organic matter recycling (Datry et al. 2018) and microbial C‐CO_2_ respiration, releasing large amounts of C to the atmosphere (Göransson et al. 2013; Kosten et al. 2018; Marcé et al. 2019; Honeker et al. 2023; Pugliese et al. 2023). This spike in heterotrophic functions following droughts has been described for continental freshwater systems in the Northern hemisphere (Datry et al. 2018; Kosten et al. 2018; Marcé et al. 2019). However, to our knowledge, evidence is lacking for small but extremely abundant systems such as tank bromeliads in the Neotropics, although they are significant sources of C emissions (see Martinson et al. 2010; Goffredi, Jang, et al. 2011; Atwood et al. 2013 for tank bromeliads), with potential implications for global C cycling projections.

## 2 Methods

### 2.1 Study Area and Experimental Setup

We conducted our experiment in French Guiana, in a lowland and largely pristine rainforest representative of the Neotropical rainforests, near the Petit‐Saut Dam (latitude: 5° 03′ 43″ N; longitude: 53° 02′ 46″ W; elevation a.s.l.: 80 m; map of forest plot location in Figure S1). This location has, on average, maximum and minimum monthly temperatures of 33.5°C and 20.5°C, respectively, and annual precipitation of about 3000 mm. There is a dry season between September and November, and a shorter dry period in March. In the study area, the average number of consecutive days without rainfall in the dry season is 26 ± 5.3 days (annual mean ± SD on daily rainfall records over the past 20 years at the Paracou weather station, 8 km away from our field site).

We experimentally manipulated the Flaming sword bromeliad *Lutheria splendens* (Bromeliaceae: Tillandsioideae), the only bromeliad species in the study area, with a density of 3558 ± 538 ind. ha^−1^ (Dézerald et al. 2018). We selected 135 plants of similar size (diameter: 70.7 ± 10.8 cm, number of leaves: 14.3 ± 1.9), and water holding capacity (V_*max*_: 187.0 ± 76.0 mL). The latter was based on measurements of vegetative traits and calculations of maximum water volume from the number of green leaves and plant diameter using established allometric relationships (Bonhomme et al. 2021). Six months before the experiment, we collected these plants in the surrounding forest area and transplanted them to the study area, planting them evenly across the forest floor of the 1 ha plot (i.e., separated by *ca*. 8 m from each other).

Our experiment followed a full‐factorial design, crossing four levels of drought duration by two levels of recolonization. Manipulations of drought duration started with the emulation of a dry phase. We used rain shelters placed over individual bromeliads (transparent plastic tarpaulin, as in Trzcinski et al. 2016), to emulate dry periods representing: (*i*) the current norm (26 days, the average maximum number of consecutive days without rainfall over the past 20 years in the area), (*ii*) IPCC average prediction in the area (current norm +40% = 37 consecutive dry days), (*iii*) a severe event typical of the area (maximum number of consecutive dry days recorded over the past 30 years = 67 consecutive days), and (*iv*) an extreme event +40% = 94 consecutive dry days. Therefore, our drought treatments in days corresponded to recent projections of the IPCC in the study area (Masson‐Delmotte et al. 2021). We also added a control treatment in which bromeliads were unmanipulated, that is, exposed to natural rainfall and macroinvertebrate colonization (baseline scenario). We staggered the start dates of our drought treatments from (*iv*) to (*i*), so that all bromeliads reached the end of their dry phase and began the rewetting phase at T_0_ simultaneously. Hence, all bromeliads experienced similar temperatures during the entire experiment, and similar rainfall during the rewetting period, regardless of the length of the drought treatment (see Figure S1a,b). This timing allowed us to match the irregular dry season with the shortest 26‐day drought treatment, while the other treatments created longer droughts over periods that would have otherwise been rainy (Figure S1). The pre‐drought conditions for all bromeliads were typical of a rainy season, where *Lutheria splendens* bromeliads at our site are filled at 51.8% ± 0.06% of their capacity (see survey in Dézerald et al. 2017). Thus, before T_0_, bromeliads essentially differed by the number of consecutive dry days they experienced (Figure S1). During the dry phase, we regularly inspected bromeliads as the amount of water progressively declined within the leaf axils, and noted the number of days a bromeliad was dry (number of “dry bromeliad days”) before reaching the rewetting phase.

At the end of the dry phase (T_0_), we first recorded the average residual water depth in the leaf axils of all bromeliads. We then removed all rain shelters simultaneously so that bromeliads underwent a rewetting phase. To start this rewetting phase, we refilled bromeliads manually with rainwater to 50% of their maximum capacity to standardize T_0_ conditions, and bromeliads then received natural rainfall over the next 60 days (Figure S1b).

At T_0_, when the rewetting phase began, we covered half of the bromeliads with a fine‐mesh net (*ca*. 100 μm) to prevent invertebrate recolonization from external source patches. In such treatments, the resilience of ecosystem functions primarily driven by invertebrates should rely on in situ tolerance (desiccation tolerance, ability to pass the drought spell at an active larval stage; see Céréghino et al. 2020) and/or in situ resistance (ability to pass the drought spell at a dormant stage; Wisnoski et al. 2019; Bonhomme et al. 2021). We added leaf litter in bromeliads covered by nets during the experiment (based on litter fall observed in control bromeliads) to compensate for the absence of natural litterfall in this treatment. The remaining bromeliads, including the control bromeliads, were not netted, so the resilience of invertebrate communities and related ecosystem functions relied on both in situ resistance/recovery and recolonization from nearby source patches.

At three time‐intervals after the drought (7, 15, and 60 days after T_0_), we sampled microbial and macro‐faunal communities, measured nutrient concentrations, and quantified three ecosystem functions (detrital decomposition, microbial respiration, and algal photosynthetic efficiency). These three time‐intervals were chosen to encompass different lengths of life cycles of invertebrates in *Lutheria splendens* bromeliads at our site (Dézerald et al. 2017). At each sampling date, we sampled five bromeliads in each drought treatment and five control, unmanipulated bromeliads. Each sampled bromeliad at any time T_7‐60_ was removed from the experiment, so replicates were independent measures as there were no repeated measures for a tank bromeliad over time. With our full‐factorial design, the 135 experimental bromeliads were thus distributed as follows: (*i*) 120 bromeliads underwent a dry and rewetting phase, i.e., 4 drought treatments (26, 37, 67, 94 days) × 2 “recolonization” treatments (net, no net) × 5 replicates × 3 rewetting durations (7, 15, 60 days after T_0_), and (*ii*) 15 bromeliads served as control (see data analysis), i.e., 5 replicates × 3 rewetting durations (7, 15, 60 days after T_0_).

### 2.2 Leaf Litter Decomposition and Associated Microorganisms

Leaf litter of *Goupia glabra* Aubl. (Goupiaceae) is commonly found in bromeliad tanks (Rodríguez Pérez et al. 2018) and is among the fastest species to decompose in French Guiana (Coq et al. 2010). Freshly‐fallen leaves were collected using nets placed under *G. glabra* trees. All leaves experienced leaching in filtered rainwater for 24 h, and were then cut into rectangular pieces, avoiding the central vein. Leaf pieces were then oven dried at 60°C for 48 h, and mass determined to the nearest 0.01 mg using an electronic balance (AB 204‐S Mettler Toledo). Leaf packs consisted of one 2 × 3 cm piece (for quantifying attached bacteria density and ergosterol, see below) and two 2 × 4 cm pieces of leaf litter (for decomposition rate), and each bromeliad received two leaf packs (in two separate water tanks within leaf axils). The mean initial dry mass of a 2 × 4 cm piece was 75.66 ± 13.07 mg (mean ± SD). At T_0_, we added leaf litter pieces by immersing each piece within separate tanks (we marked the corresponding leaf with colored tape), so that leaf pieces experienced micro‐conditions close to natural leaf litter in tank bromeliads. These four 2 × 4 cm pieces in each bromeliad were used to estimate decomposition at 7, 15, and 60 days after T_0_ (by averaging their mass at T_0_ *and* T_final_). Hence, initial and remaining leaf mass (*m*_0_ and *m*_T_, respectively) at each of those three sampling dates T were used to estimate litter decomposition rate *k* (d^−1^) using the exponential decay model, as follows:

(1) $$k=–\ln\;\left(m_{T}/m_{0}\right)/T$$

The 2 × 3 cm pieces were used to assess the density of bacteria attached to leaf litter (cells per mg dry mass) and its ergosterol content (μg per mg dry mass, a proxy of fungal biomass). We quantified bacterial abundances at the surface of the leaves using flow cytometry after sonication of the leaf pieces (see below). To estimate the fungal biomass developing in the leaf litter, we analyzed ergosterol contents obtained by lipid extraction and HPLC analysis following Gessner and Schmitt (1996).

### 2.3 Invertebrate Communities

After collecting the leaf litter pieces, we carefully sampled each bromeliad by pipetting out all the contained water, detritus, and aquatic organisms, using 10 mL micropipettes with the end trimmed to widen the aperture (Jocque et al. 2010). To maximize sampling efficiency, we sucked the water in and out of the pipette three times before extracting it (Rodríguez Pérez et al. 2018). We sieved the extracted contents on a 150 μm mesh sieve to retain the largest particles and macroinvertebrates. We identified macroinvertebrates in the laboratory to species or morphospecies and counted them. We assigned taxa to functional feeding groups (FFGs, namely predators, shredders, scrapers, filter‐feeders, or deposit‐feeders) based on prior knowledge of their trophic ecology (Céréghino et al. 2018). We calculated per capita dry masses by pooling a sufficient number of individuals by taxon, oven‐drying them (24 h, 60°C), determining their mass to the nearest μg, and then dividing this mass by the number of individuals (Dézerald et al. 2017). Then, we calculated FFG biomasses from per capita dry masses and abundance data (Table S1).

In the field, we also collected subsamples of water and immediately brought these to the laboratory in a cooler for subsequent analyses of nutrient concentrations, microbial respiration, free bacteria density, and algal photosynthetic efficiency (see below).

### 2.4 Nutrient Concentrations

We took 15 mL subsamples of bromeliad water to analyze concentrations of dissolved organic carbon (DOC), total nitrogen, and total phosphorus (DOC, N_tot_, P_tot_). Specifically, we analyzed DOC and N_tot_ with a Shimadzu TOC analyzer and a Thermo Scientific Flash 2000 elemental analyzer, respectively. We analyzed dissolved P_tot_ with a Secoman UVI light XTS spectrophotometer.

### 2.5 Microbial Respiration and Bacterial Counting

We quantified microbial C‐respiration of water contained in tank bromeliads using the MicroResp method, a microplate‐based respiration system allowing CO_2_ release measurements from microbial communities (Campbell et al. 2003). We measured C respiration from water rather than from organic matter since the volume of water held by tank bromeliads is well‐documented (Martinson et al. 2010; Goffredi, Kantor, and Woodside 2011), allowing us to scale up experimental observations to regional estimates of C respiration from tank bromeliads (e.g., see Martinson et al. 2010).

Briefly, we added 1 mL of bromeliad water filtered at 100 μm to a 96‐deep‐wells microplate, allocating 4 wells per true replicate. Then, we sealed the 96‐deep‐well microplate with a 96‐well detection microplate containing agar gel and cresol red as indicator dye (Campbell et al. 2003), and incubated the plates at 25°C in the dark for 18 hours. We measured discoloration of the indicator gel using spectroscopy at 570 nm with a multi‐plate spectrophotometer (TriStar LB 941 microplate reader Berthold Technologies). We normalized the absorbance values at a given time by the initial absorbance values, enabling us to calculate the percentage of CO_2_ released from each well (Campbell et al. 2003). Specifically, we averaged values for the four technical pseudo‐replicates and subtracted control values. As a control, we used a bromeliad water sample filtered at 0.2 μm (GF/F Whatman filter), freed of its microorganisms. We expressed microbial respiration in μg of C‐CO_2_ released per hour and by mL of water.

Finally, we counted free‐living and attached bacteria using a FACSCalibur flow cytometer (Becton Dickinson). For attached bacteria, we placed the 2 × 3 cm piece of leaf litter (one per bromeliad) in TE buffer (10 mM Tris, 1 mM EDTA) and fixed it with 2% paraformaldehyde before analysis. Then we incubated samples with sodium pyrophosphate (10 mM) and sonicated each sample for 30 s using a sonication bath, to disaggregate cells. We centrifuged the bacterial suspensions at 800 g for 60s, diluted the supernatants 10‐fold, and stained them with SYBR Green I (Molecular Probes). After this step, we counted attached bacterial cells with a BD FACSCalibur flow cytometer (15 mW at 488 nm, Becton Dickinson, U.S.A.). We counted free‐living bacteria from 1 mL subsamples of bromeliad water after staining with SYBR Green I.

### 2.6 Microalgal Photosynthetic Efficiency

Microalgal production in bromeliads is low in rainforest understories, such as at our study site, due to canopy cover limiting the incident radiation (Brouard et al. 2012). Thus, microalgal counts can be associated with large errors, so we used the more sensitive quantum yield measures of the photosystem II (hereafter ‘PS II’) as a proxy for algal photosynthetic activity. PSII quantum yield provides a measure of the photosynthetic efficiency of microalgal communities, and is therefore linked to their population physiological status (Smith et al. 2006). We quantified photosynthetic efficiency of microalgae from three 3 mL subsamples of water per bromeliad, filtered on a GF/F Whatman filter (0.7 μm), to recover the microbial community. We used a Phyto‐PAM fluorometer (Walz, Effeltrich, Germany) to measure the fraction of the absorbed quanta used for photosynthetic electron transport in the PS II. We performed all measurements at the field station, in a dark chamber according to Wilken et al. (2013), and subsequently averaged technical pseudo‐replicates.

### 2.7 Data Analysis

The baseline used to evaluate ecosystem resilience is often the pre‐disturbance state (McCrackin et al. 2017). However, in highly dynamic systems, it is advisable to use an undisturbed reference at the time of sampling (Ingrisch and Bahn 2018). This is relevant to the dynamic tank bromeliad biota and ecosystem (see Dézerald et al. 2017). We expressed, as a percentage of deviation *P%*(*f*)_*ij*_, each function or ecological state variable *f* of any tank bromeliad *ij*. To calculate this percentage deviation, we divided the function variable *f*_*d*T_ for a bromeliad with drought *d* at a given rewetting time T (namely 7d, 15d, and 16d) by μ_1/2_(*fc*T), that is, the median value (μ_1/2_) for that same function *f* in the corresponding control bromeliads *c* for that same sampling time T:

(2) $$P\%{\left(f\right)}_{\mathrm{ij}}=f_{dT}/\mu_{1/2}\left(f_{cT}\right)$$

We used the median rather than the mean here to avoid the influence of extreme values in controls.

As the analysis of resistance (i.e., short‐term response of multifunctionality immediately following the drought spell) had only one time period (i.e., at 7d of rewetting), we used a different approach for this treatment: we analyzed the 7 days rewetting responses in their natural units, and compared ecosystem function values between drought treatments and unmanipulated bromeliads, using ANOVA followed by pairwise Tukey's HSD tests. These, and all further statistical analyses, were done with R version 4.3.2 (R Development Core Team 2023).

We assessed the dynamics of single ecosystem functions during the rewetting phase with linear regressions of percent deviations *P%(f)ij* (see Equation 2) relative to unmanipulated bromeliads (i.e., recovery potential; Ingrisch and Bahn 2018). The slope of these regressions provides an estimate of the percentage of change relative to controls by day of rewetting (% d^−1^). We also considered changes in slope and intercept between open and netted bromeliads, thus evaluating the effect of invertebrate recolonization from nearby source patches (Bonhomme et al. 2021).

Ecosystem multifunctionality has often been measured as the average of *z‐*transformed ecosystem functions (Maestre et al. 2012). This averaging approach, despite being straightforward, has some limitations (see Byrnes et al. 2014; Bradford et al. 2014). By contrast, the multiple‐thresholds approach, i.e., the number of functions exceeding varying thresholds of the maximum values of each function, is so far the most comprehensive way to express multifunctionality (Byrnes et al. 2014). We therefore approached multifunctionality with both the multiple‐thresholds approach and by examining ecosystem functions in isolation, as suggested in Byrnes et al. (2014). We first computed the number of functions exceeding thresholds comprised from 1% to 99% of their maximal values (i.e., the average of the three highest values for each function). Then, for each threshold, we used generalized linear models (GLMs) with quasi‐Poisson errors and the identity link to compute the slopes of the linear relationships between dry bromeliad days and multifunctionality and their 95% confidence intervals, with the R package ‘*multifunc*’ (Byrnes et al. 2014). We implemented this procedure for each rewetting duration (7d, 15d, and 60d). By doing so, we expressed each slope as the change in number of functions per dry bromeliad day (*n* functions d^−1^) at each rewetting stage, hypothesizing that the effects of drought intensity would attenuate during the rewetting of the system. In addition, we visualized shifts in multifunctionality dominance with drought intensity and rewetting using a normalized (*z*‐transformed) Principal Component Analysis (PCA) on the matrix of baseline‐normalized ecosystem functions (“*ade4*” R package; Thioulouse et al. 2018). We tested the effects of drought intensity (26d, 37d, 67d, 94d), rewetting (7d, 15d, 60d after T_0_) and recolonization (net, open) treatments and the drought × rewetting and rewetting × recolonization two‐way interactions on the same Euclidean C‐related multifunctionality dominance matrix with a Permutational Multivariate Analysis of Variance (PERMANOVA; “*vegan*” R package; Oksanen et al. 2007). This multivariate analysis was intended to test shifts in the dominance of functions with the experimental treatments (Giling et al. 2019), and therefore was complementary to the analyses of multifunctional thresholds and individual functions taken in isolation (Byrnes et al. 2014).

To test how abiotic and biotic ecological states would mediate the resilience dynamics of multifunctionality following drought in our experimental tank bromeliads, we followed the general framework suggested by Giling et al. (2019). We performed a structural equation model (SEM) analysis (‘*piecewiseSEM*’ R package; Lefcheck 2016), based on our a priori meta‐SEM (see Figures S2 and S3). This SEM was used to quantify the direct and indirect effects of our treatments (drought, rewetting, recolonization, and two‐way interactions) on ecological state variables (abiotic and biotic variables) and ecosystem functions (decomposition, respiration, and photosynthetic efficiency). After building a full model, we performed step‐by‐step backward variable selection of the least non‐significant variables for each response in the model, and applied this procedure until all paths retained in the SEM were significant. In other words, if an ecological state variable was not a significant predictor, it was excluded from the SEM. After having checked for any missing link with d‐separation tests, we used a Chi‐square test to assess the goodness‐of‐fit of the retained model (Lefcheck 2016). We further conducted a path analysis on the SEM results. For this purpose, we bootstrapped our retained SEM model a thousand times and calculated direct and indirect effects using the ‘*semEff*’ R package.

To finish, we used our experimental measurements of microbial respiration (in μg C‐CO_2_ mL^−1^ h^−1^) to scale up tank bromeliad C‐emissions following droughts at the spatial scale of Amazonian forests, under various realistic scenarios (see detailed methodology and results in Supporting Information, Appendix S2).

## 3 Results

### 3.1 Resistance of Single Functions Following Drought

**FIGURE 1:**
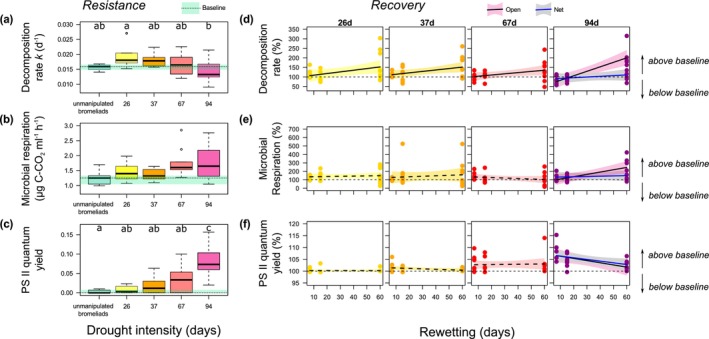
Resistance and recovery dynamics of functions taken in isolation. (a–c) Short‐term responses of each ecosystem function to drought duration (7 days after rewetting) of (a) litter decomposition rate k (d−1), (b) microbial C‐respiration (μg C‐CO2 mL−1 h−1), and (c) algal photosynthetic efficiency (quantum yield values of the PSII). Categories represent both drought intensity (26, 37, 67, and 94 days without rainfall; yellow to pink boxplots) and unmanipulated bromeliads (in turquoise). Different letters denote significant pairwise differences among treatments. One outlier is not shown (panel b, 37d of drought; 8.602 μg C‐CO2 mL−1 h−1). (d–e) Dynamics of decomposition (d), microbial C‐respiration (e), and algal photosynthetic efficiency (f) in bromeliads submitted to drought scenarios (from left to right; 26, 37, 67, and 94 days without rainfall) for each sampling date following drought (rewetting phase, in days). Each ecosystem function is expressed as a percentage of deviation from median values observed in unmanipulated bromeliads (%). Note that the y‐axis scale differs substantially between functions. Regression lines represent the dynamics of each ecosystem function during the rewetting phase, with their 95% confidence bands. Solid regression lines show significant slopes, while dashed regression lines show non‐significant slopes. Blue lines and grey bandwidth are given for the treatment with a net to prevent colonization (plotted for cases where a rewetting × recolonization interaction was significant).

Ecosystem functions taken in isolation showed contrasting resistance patterns to drought treatments, immediately (7 days) after rewetting (Figure 1a–c). Leaf litter decomposition rate was significantly affected by drought intensity (*F*_4,38_ = 3.53; *R* ^2^ = 0.27; *p* = 0.0152; Figure 1a). Maximal decomposition occurred in bromeliads that underwent 26d without rainfall, and then declined 28% as drought intensity increased to 94d (Tukey HSD test; *p* = 0.0098; Figure 1a). Short‐term responses of microbial respiration did not differ significantly among treatments (Figure 1b, *F*_4,40_ = 0.54; *R* ^2^ = 0.05; *p* = 0.7093). Photosynthetic efficiency (quantum yield of PSII) increased significantly with drought intensity (*F*_4,40_ = 11.94; *R* ^2^ = 0.54; *p* < 0.0001; Figure 1c), and was twice as high after 94d of drought than in control bromeliads (Tukey HSD test; *p* < 0.0001) and departed significantly from all other drought treatments (Tukey HSD tests always *p* < 0.01; Figure 1c).

### 3.2 Dynamics of Single Ecosystem Functions During the Rewetting Phase

Ecosystem functions also showed contrasting trajectories during the rewetting phase, depending both on drought intensity and the colonization treatment (Figure 1d–f). Considering all drought durations, decomposition increased by 44% from 7d to 60d of rewetting, compared to the baseline (*F*_1,116_ = 26.28; *R* ^2^ = 0.28; *p* < 0.0001; Figure 1d). However, this increase in decomposition was amplified at extreme drought duration (94d) coupled with colonization: during rewetting, litter decomposition increased 15‐times faster in open bromeliads than in the netted ones (*F*_1,26_ = 13.33; *R* ^2^ = 0.51; *p* = 0.0012; Figure 1d). Microbial respiration increased by 59% during the rewetting phase after the most extreme drought of 94d (*F*_1,26_ = 6.43; *R* ^2^ = 0.17; *p* = 0.0176; Figure 1e), and this increase was 66‐times faster in open bromeliads where invertebrate recolonization was allowed (*F*_1,26_ = 6.06; *R* ^2^ = 0.16; *p* = 0.0208; Figure 1e). By contrast, photosynthetic efficiency showed the reverse pattern: it was unaffected by rewetting for droughts of 67d or less, but decreased by 4% during the rewetting period after 94d of drought (*F*_1,27_ = 10.21; *R* ^2^ = 0.25; *p* = 0.0035; Figure 1f). This return to a reference state of photosynthetic efficiency was 1.2‐fold faster for open than for netted bromeliads (*F*_2,26_ = 5.04; *R* ^2^ = 0.22; *p* = 0.0141; Figure 1f).

### 3.3 Multifunctionality in the Face of Drought Intensification

**FIGURE 2:**
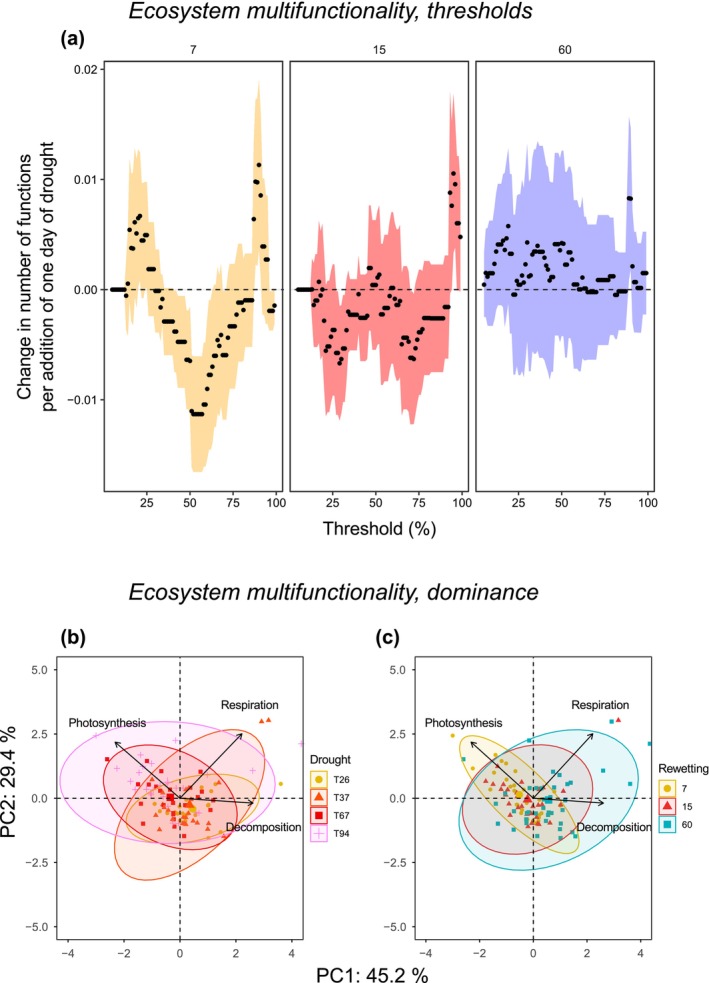
Ecosystem multifunctionality dynamics. (a) Linear trends of drought intensification (number of dry days) on multifunctionality (change in number of functions per addition of one day of drought) for each rewetting date (7d, 15d, and 60d) and multifunctionality threshold considered (from 1% to 99% of the maximum of each function expressed as relative to the unmanipulated bromeliad baseline). Colored areas are 95% confidence intervals for each computed slope, and panels from left to right are given for 7d (yellow), 15d (red), and 60d (blue) of rewetting following drought. (b–c) Distribution of bromeliads in the multivariate space of baseline‐normalized C‐related ecosystem functions (normalized Principal Component Analysis, PCA on percentage deviation values from unmanipulated bromeliads). The first and second PCA axes captured 45.2% and 29.4% of variance in the dataset, respectively (74.6% total). (b) Effect of drought intensity (yellow: 26d; orange: 37d; red: 67d; pink: 94d) on decomposition, photosynthetic efficiency, and respiration. (c) Effect of rewetting on ecosystem functions (yellow: 7d after the drought has passed; red: 15d; blue: 60d). Ecosystem functions are represented as vectors.

Multifunctionality relative to the baseline was weakly influenced by drought and rewetting time, a pattern that stayed roughly consistent across varying thresholds (Figure 2a). Nevertheless, drought showed negative effects on multifunctionality after just 7 d of rewetting, especially when we considered thresholds in the range 40%–60% (at a 40% threshold; *F*_1,36_ = 4.18, *p* = 0.0484; 50% threshold; *F*_1,36_ = 17.55, *p* = 0.0002, and 60% threshold; *F*_1,36_ = 5.08, *p* = 0.0303, respectively; Figure 2a). Multifunctionality tended to return progressively to a baseline state, as linear trends generally overlapped zero by 15 d, and by 60 d, they tended to be slightly positive (Figure 2a). Positive and negative effects of drought on multifunctionality at any threshold were weak, as they did not exceed ±0.02 functions by additional day of drought, with 95% confidence intervals largely overlapping zero (Figure 2a).

The PCA showed that baseline‐normalized functions covaried weakly, and 75% of the variance was explained by the first two principal components (Figure 2b,c). Leaf litter decomposition and microbial respiration responses (both heterotrophic functions) were weakly and positively correlated with each other (Pearson's *r* = 0.20, *p* = 0.051). Microbial respiration and photosynthetic efficiency responses were not correlated (Pearson's *r* = −0.12, *p* = 0.23), but leaf litter decomposition and photosynthetic efficiency showed a weak negative correlation (Pearson's *r* = −0.22, *p* = 0.032).

The PERMANOVA analysis showed that drought and rewetting durations induced shifts in multifunctionality (*total R* ^2^ = 0.26; Table 1). After a drought emulating the current norm (26 consecutive days), multifunctionality dominated by heterotrophic functions was above the controls (Figure 2b; Table 1). As drought intensity increased up to 94d, we observed a shift in multifunctionality, with the dominance of photosynthetic efficiency (Figure 2b; Table 1). This pattern reversed during the rewetting phase, as by 60d of rewetting, ecosystem multifunctionality was dominated by heterotrophic functions (Figure 2c; Table 1).

**TABLE 1:** Permutational Multivariate Analysis of Variance (PERMANOVA) statistics of the effects of treatments on the multivariate space of the three ecosystem functions (z‐scores). Total *R* ^2^ values included all factors, whether they were significant or not (*R* ^2^ = 0.26). *R* ^2^ value for significant factors only was *R* ^2^ = 0.20.

Variable	Df	SS	*F*	*R* ^2^	*P*
Drought	3	34.54	4.75	0.12	0.001
Rewetting	1	24.75	10.20	0.08	0.001
Recolonization (net vs. open)	1	4.30	1.77	0.01	0.135
Drought × rewetting	3	12.72	1.75	0.04	0.085
Recolonization × rewetting	1	2.32	0.96	0.01	0.411
Residuals	90	218.375		0.74	

### 3.4 Abiotic and Biotic Drivers of Multifunctionality Following Droughts

**FIGURE 3:**
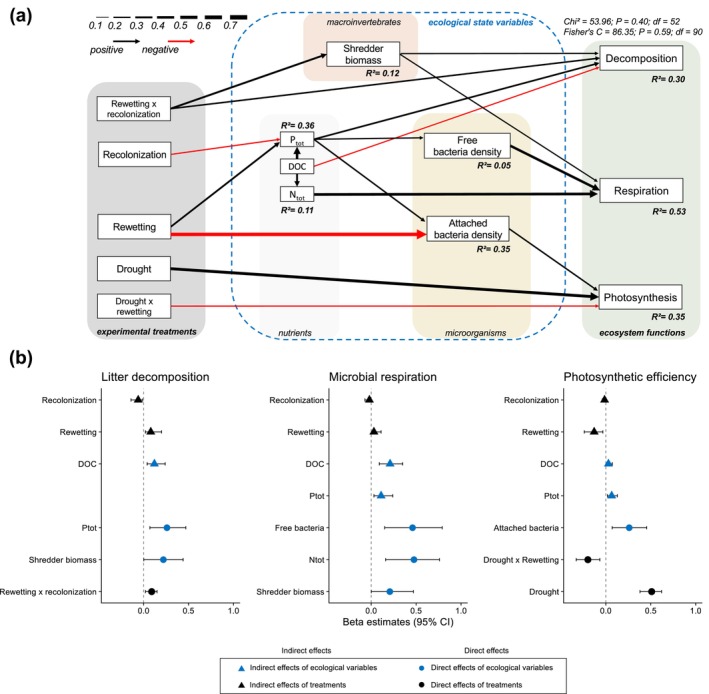
(a) Final structural equation model (SEM) of multifunctionality resilience dynamics following drought. Only significant paths are depicted here. The size of the arrows is proportional to standard beta coefficients (full statistics reported in Table S2). Red lines are negative relationships, and black lines are positive ones. *R* ^2^ values are given for each response variable. (b) Significant direct and indirect effects computed from the SEM (standard beta coefficients and their 95% confidence intervals). We distinguished amongst direct versus indirect effects (circles vs. triangles, respectively), and between the effects of ecological state variables versus the effects of the treatments themselves (blue vs. black, respectively) on single ecosystem functions.

The retained SEM showed that the post‐drought dynamics of multifunctionality were influenced by direct and indirect relationships among nutrients, bacterial densities, and the biomass of shredders (Figure 3a). As expected, changes in shredder biomass positively affected litter decomposition. Microbial respiration following droughts was increased by free‐living bacteria density, and the latter was positively affected by changes in total dissolved phosphorus during the rewetting phase (Figure 3a). Photosynthetic efficiency responses were primarily driven by direct positive effects of drought, negative effects of rewetting duration, and direct positive effects of the density of attached bacteria to leaf litter (Figure 3a).

The dynamics of shredder biomass, free bacteria and attached bacteria density, and the dynamics of DOC, P_tot_, and N_tot_ concentrations can be seen in Supporting Information (Figures S4 and S5). We also show that using the realized number of days of drought by bromeliad reflected our drought treatments (*R* ^2^ = 0.84; *F*_4,130_ = 173.6; *p* < 0.0001; Figure S6). Although all FFGs but shredders were excluded in the final SEM (Figure 3), the responses of other functional feeding groups (FFGs) to drought, rewetting, and recolonization treatments revealed distinct patterns (Figure S7 and Table S3). The post‐drought dynamics of shredder biomass (Figure S4a) tended to be similar to those of litter decomposition (Figure 1d): a weak general increase over the rewetting period for droughts from 26 to 67d (Figure S4a), but a net positive departure above the baseline following 94d of drought. This was particularly marked for the treatment where recolonization was allowed (Figures S4a and S8). This large increase could result from the complete reduction of predator biomass following the 94d drought, and the inability of predators to recover to baseline conditions during rewetting (Figure S8).

Unlike shredder biomass, attached bacterial density was positively affected by drought and negatively affected by rewetting, a pattern that was consistent with changes in photosynthesis efficiency (Figure 1f). The post‐drought dynamics of N_tot_ and P_tot_ were positively correlated with those of DOC (Figure 3a), with P_tot_ levels increasing with rewetting, especially following 94 d of drought (Figure S5). Overall, the indirect and direct effects of nutrients on the three functions were consistently positive (Figure 3). The final SEM (Figure 3) excluded ergosterol concentrations (a proxy for fungal biomass) and the biomass of other functional feeding groups (FFGs) except shredders, as these variables did not significantly explain any paths in the model. Overall, the SEM analysis emphasized that the post‐drought dynamics for single functions were directly and positively coupled to multiple different groups of organisms, with a direct positive effect of shredder biomass on decomposition and microbial respiration, a direct effect of free bacteria density on microbial respiration, and a direct effect of attached bacteria densities on photosynthetic efficiency (Figure 3).

## 4 Discussion

We subjected replicated, individual bromeliad ecosystems to drought intensities ranging from the ambient norm (26d) to 3.6 times the current average number of consecutive days without rainfall in the study area (94d). Overall, intensifying droughts only slightly impaired ecosystem multifunctionality in the first 7 days of rewetting. Multifunctionality then recovered to baseline levels, showing at most weak effects of drought on multifunctionality, which suggests insurance mechanisms among drivers of individual functions.

After the 94‐day drought passed, we observed a shift in multifunctional recovery marked by two phases. In the short term (7 days following drought), photosynthesis showed values above baseline conditions, which recovered to baseline levels during rewetting. Although netted bromeliads recovered faster than open bromeliads, the fine‐mesh net may have slightly decreased light availability in this treatment, constituting a potential source of bias. Litter decomposition and microbial respiration showed a different pattern. Both heterotrophic functions were only weakly affected by drought intensification in the short term, but increased sharply above baseline levels following the most extreme drought (94d), and did not return to baseline levels within the 60 days of rewetting. Interestingly, this deviation of heterotrophic functioning above baseline levels was even faster when recolonization of invertebrates was permitted, as in natural conditions (open bromeliads). Our SEM analysis showed how this shift in heterotrophic functioning was driven by positively covarying abiotic (i.e., nutrients) and biotic factors (bacterial density and shredder biomass). Overall, our study on the ecosystem multifunctionality responses of tank bromeliads to drought intensification in the Neotropics underscores the importance of adopting a meta‐ecosystem perspective that incorporates the dispersal of organisms, biotic interactions, and fluxes of matter in ecosystems (Thrush et al. 2009; Massol et al. 2017).

### 4.1 Mechanisms Underpinning Ecosystem Multifunctionality Dynamics

Photosynthetic efficiency peaked immediately after the longest drought treatment. The release of nutrients from dead organic matter during the rewetting phase could have boosted photosynthetic efficiency in the short term, a mechanism also observed in drying rivers (Timoner et al. 2014). Algae are also capable of dormancy, a bet‐hedging life‐history strategy allowing to cope with environmental unpredictability (Wisnoski et al. 2019). This post‐drought peak in photosynthesis may be due to the physiological peculiarities of algal cells released from dormancy, in addition to the availability of carbon and nutrients released from dead organic matter upon rewetting (Timoner et al. 2014; Sabater et al. 2016). Alternatively, this peak may also be attributed to algal community turnover, where drought‐tolerant species with photosynthetic capacities typical of pioneer species may have dominated the early succession of assembly (Sabater et al. 2016). A lagged priming effect (from both algae and bacteria), where labile organic carbon from microbial exudates (i.e., DOC) increases the mineralization of recalcitrant carbon pools (i.e., leaf litter), could have occurred during the rewetting phase, creating positive feedbacks which could have reinforced the large increase of heterotrophy above baseline levels (Danger et al. 2013; Scheffer and Carpenter 2003). The alternation of dominance between ecosystem functions, with photosynthesis dominating multifunctionality after the extreme drought, followed by a strong but delayed increase of heterotrophy above baseline levels at 60 days of rewetting, suggests multifunctional instability of the system following disturbance intensification (Scheffer and Carpenter 2003; Thrush et al. 2009). Future investigations will tell us whether heterotrophic functions can undergo a longer‐term shift in response to harsh droughts in tank bromeliads, as we stopped our observations at 60 days following drought events.

To understand the mechanisms underpinning multifunctional responses to global changes, covariations among abiotic and biotic factors need to be taken into account (Maestre et al. 2010; Giling et al. 2019). Microbial respiration and litter decomposition trajectories were positively mediated by nutrient dynamics, nutrients that were probably re‐mineralized during the rewetting of detritus (i.e., broken cells, decomposing metazoans, and organic matter). Since bacteria and algae exhibit short generation times (hours to days), we expected that the functions they directly animate (microbial respiration and photosynthetic efficiency, respectively) would express fast dynamics after severe to extreme drought events. Accordingly, we observed an immediate positive effect of drought intensification on photosynthetic efficiency at 7 days of rewetting following droughts. This might have contributed to a delayed priming effect on litter decomposition. For the same reason, however, we may expect that an algal priming effect would have induced an immediate effect on bacterial densities and on microbial C respiration. While we observed that attached bacterial densities and photosynthesis efficiency followed roughly similar dynamics (Figure S4), we rather observed a lagged increase of microbial C respiration during the rewetting stage. As expected, the release of N, P and DOC in the water column during rewetting favored bacterial growth and respiration rates (Figure 3b) (Smith et al. 2006). This release of nutrients could have also increased the palatability of leaf litter for shredders, therefore reinforcing the lagged increase in litter decomposition. The excretion of nutrients and fine particulate organic matter (FPOM) from shredding activity may have then increased microbial respiration in a positive feedback (Figure 3). Finally, the positive link between changes in bacterial densities attached to litter and photosynthesis efficiency from water samples may be the result of a positive, unobserved covariation between this group of bacteria and the algae in the water column. Microalgae can mediate important feedbacks within the microbial food web and influence heterotrophic processes such as CO_2_ respiration and decomposition. For instance, they can release 20%–40% of their photosynthetic products into their surrounding environment. These exudates often comprise carbohydrates, such as polysaccharides and amino acids, providing energy to bacteria. As such, bacterial abundance is often correlated with microalgal density and activity (Wyatt and Turetsky 2015; Hamard et al. 2025).

Litter decomposition depends mostly on the biotic activity of heterotrophic microorganisms (bacteria and fungi) and invertebrate shredders (Gessner et al. 2010). Whilst our SEM showed that bacterial density and shredder biomass positively drove the dynamics of microbial respiration and litter decomposition through a combination of direct and indirect effects, these two groups of organisms did not contribute equally to each function. Changes in shredder biomass relative to control bromeliads was positively and directly linked to changes in both decomposition and microbial respiration (Figure 3). Bacterial density in the water column was rather unaffected by drought intensity, but it correlated positively and directly with microbial respiration dynamics. Further, we observed a positive coupling between the changes in attached bacteria density and the changes in photosynthetic efficiency, albeit this link stayed largely unexplained to us. Altogether, the dynamics of different types of organisms were coupled to the individual dynamics of the three ecosystem functions. As these ‘organism–function’ couplings responded to drought and rewetting often in lagged and opposite directions, net effects of drought intensity on multifunctionality were weak. To us, these compensatory mechanisms may be the most general rationale to explain why we observed virtually no effect of drought intensification on ecosystem multifunctionality using the threshold approach, a mechanism that we may refer to as multifunctional insurance (Loreau et al. 2021; Eisenhauer et al. 2024).

Only photosynthetic efficiency followed a “classic” post‐disturbance deviation and recovery to the initial baseline. Litter decomposition trajectories followed an immediate suppression by the harshest drought and then a transient overcompensation up to 60 days of rewetting following the 94d drought treatment. Microbial respiration was unaffected by drought intensity in the short term (showing resistance), but, as litter decomposition, showed a transient increase up to the baseline until 60 days of rewetting. In addition to the detailed analysis of abiotic and biotic drivers that we developed here, further investigations on tank bromeliads would help in reaching a comprehensive understanding of the mechanisms leading to a diversity of responses to drought by individual functions, and of potential compensatory dynamics within an ecosystem multifunctionality framework.

Our experimental results relate to meta‐ecosystem theory (Massol et al. 2017). The increase in litter decomposition following the harshest 94d drought was 15‐fold higher in open than in netted bromeliads. Shredders' biomass followed a similar pattern (Figure S8). In natural conditions (open bromeliads), the 4‐fold slower and incomplete recovery of invertebrate predators compared to their shredder prey following droughts (Ruiz et al. 2022) may have released invertebrate shredders from predation pressure relative to unmanipulated bromeliads (see Figure S8). Following tri‐trophic food chain theory, a release from predation would explain the 2‐fold increase of shredder biomass above baseline levels at 60 days of rewetting (Figure S8), thus reinforcing the burst in litter decomposition in open bromeliads in the extreme drought scenario. Further experiments of this type are needed to develop a mechanistic understanding of how multifunctionality resilience to drought is driven by the dynamics and stability of multi‐trophic communities. From a phenomenological standpoint, our experiment agrees with the conclusion of a recent study that the dispersal of organisms among patches is an essential component of multifunctional resilience to drought (Séguigne et al. 2024b).

### 4.2 Resilience of the Meta‐Ecosystem

Our experiment, which manipulated recolonization for entire ecosystems along a drought intensification gradient, challenges the expectations for dispersal—stability relationships in meta‐ecosystems. Following the dispersal–induced stability (D‐IS) hypothesis, we may expect dispersal to stabilize ecosystem functions in the face of disturbance (Pedersen et al. 2016; Massol et al. 2017; Anderson and Fahimipour 2021), but we found the opposite pattern. The heterotrophic shift observed after the 94d drought was reinforced by the differential recolonization rates of shredders and predators, two functional groups known to have synergistic effects on nutrients and C‐cycling in tank bromeliads (Ngai and Srivastava 2006). Thus, to fully understand multifunctional resilience to drought, we need to integrate species interactions within (e.g., competition, predation, complementarity) and among (e.g., spatial and spatio‐temporal dispersal through dormancy; Wisnoski et al. 2019) multi‐trophic tank bromeliads. Poor hydrological conditions for predator invertebrates, if they become the norm, could destabilize food webs in tank bromeliads, causing a shift from top‐heavy to bottom‐heavy pyramids (Romero et al. 2016; McCauley et al. 2018), with potentially strong, but not yet studied, large‐scale effects on C cycling.

We show here that recolonization dynamics by macroorganisms following disturbance play an important role in the trajectories of ecosystem C‐related multifunctionality after extreme drought events. However, the real‐world outcomes of these dynamics are difficult to predict (Scheffer and Carpenter 2003; Thrush et al. 2009). For example, to extrapolate our conclusions to real‐world conditions, we would need to account for the spatial extent and patchiness of droughts in the forest landscape. Our local experiment inherently assumed that drought events, even extreme droughts expected to dry out bromeliads regionally, would be patchy locally, as obviously we did not manipulate droughts at the regional scale. By allowing tank bromeliads to be recolonized from undisturbed, adjacent plants, we simulated a scenario where the drought could have been heterogeneous at the forest scale. Indeed, the effects of moderate droughts could be relatively patchy at the forest scale, owing to heterogeneous micro‐climatic conditions in terms of evaporation rate, stemflow, and throughfall (Bialkowski and Buttle 2015). However, droughts are predicted to have dramatic effects when both extreme and homogeneous at the regional scale, as tended to occur in Amazonian forests during major drought events in 2005, 2010, and 2015/16 (Papastefanou et al. 2022). Such regional‐scale, extreme climatic events may totally suppress any short‐term recolonization of functionally important species. With our experiment, we simulated this regional‐scale scenario in netting tank bromeliads after a 94‐day extreme drought, preventing any recolonization from the regional pool.

A valuable next step would be to explore how repeated droughts could induce legacy effects that influence multifunctionality during subsequent droughts (Müller and Bahn 2022), and tank bromeliads would be ideal for such tests. Additionally, our study was limited not only in space—since droughts occur regionally—but also in time. While the 94‐day drought was extreme, extending the rewetting phase (e.g., to 6–12 months) could provide deeper insights, as the increase in heterotrophic functions had not yet stabilized after 60 days.

### 4.3 Perspectives for C‐Cycling at the Regional Scale

Given the importance of Neotropical forests for biodiversity and C‐cycling, and the extremely fast environmental and climate changes occurring in this region, combining observational, experimental, and modelling approaches will be essential for predicting the resilience—or the lack thereof—of C‐related multifunctionality in these systems, their contribution to global C‐cycling (Doughty et al. 2015), and potential feedbacks on the global climate. Tank bromeliads, because of their high densities (Downing et al. 2006) and large reliance on allochthonous C inputs, are known to contribute substantially to global carbon emissions, notably in being special habitats for archaeal communities involved in C‐CH_4_ emissions (Goffredi, Jang, et al. 2011; Brandt et al. 2015, 2017), at an estimated rate for the Neotropics averaging to 1.2 Tg of C‐CH_4_ yr.^−1^ (Martinson et al. 2010). Indeed, our measurements of C‐CO_2_ respiration provide useful data to scale up globally C‐CO_2_ loss from tank bromeliads to the atmosphere in response to droughts. Here, our intention was not to provide accurate estimates of C‐CO_2_ emissions from bromeliads in the Neotropics. Actually, our upscaled estimates were intended to shed light on the magnitude and range of potential shifts in C‐CO_2_ loss with drought intensification.

Based on our estimates, with droughts ranging from the current norm (26d) to extreme droughts (94d), and covering from 5% to 50% of the Neotropical forests area, tank bromeliads could emit, in addition to baseline levels, 0.2 Pg C‐CO_2_ yr.^−1^ (95% confidence intervals: 0.1–0.3) for 5% of drought coverage area, 0.4 Pg C‐CO_2_ yr.^−1^ (0.2–0.6) 95% CI for 10% of drought coverage, and 2 Pg C‐CO_2_ yr.^−1^ (1.1–2.9) 95% CI for 50% of drought coverage area, respectively (Figure S9; Appendix S2). Such increases in C‐loss fall within the range of C‐losses observed from three recent regional disturbances: (*i*) reduced tree photosynthesis and increased tree mortality during the severe Amazonian droughts of 2005, 2010, and 2015/16 (from 0.2 to 1.6 Pg C yr.^−1^; Phillips et al. 2009; Gatti et al. 2014; Doughty et al. 2015; Yao et al. 2023); (*ii*) megafires (0.8 Pg C for the year 2024; Bourgoin et al. 2025); and (*iii*) deforestation (0.34 Pg C yr.^−1^; Bullock and Woodcock 2021). Such global change factors are part of positive feedback loops with the global climate, and our results suggest that tank bromeliads should be included in changes in global C budgets from Neotropical forests in response to global changes (Cramer et al. 2004; Doughty et al. 2015; Nottingham et al. 2019).

However, we caution readers in interpreting the point estimates of C‐loss we report here, along with their associated uncertainties propagated via Markov Chain Monte Carlo (MCMC) estimation methods (see the sensitivity analysis in Supporting Information, Appendix S2). These estimates are based on *ex situ* respiration measurements and rely on key assumptions, such as tank bromeliad densities and water storage capacities. Therefore, in situ measurements, accounting for gas exchanges at the water–air interface, day‐night phases, seasonality, and covering the range of sizes of the species‐diverse tank bromeliads, will be needed to refine the estimates we provide here. This could be ideally done using floating chambers adapted to the generally small size of the system (Martinson et al. 2010) or the use of the headspace technique (Atwood et al. 2013).

Future attempts to upscale the multifunctional responses to drought intensity in tank bromeliads will benefit from improved accuracy of bromeliad abundance estimates and spatial distribution in the Neotropics, using high‐resolution satellite imagery (Mullen et al. 2023). This knowledge, combined with in situ experimental manipulations of droughts and resilience measures of key functions related to major element cycles, as reported here, will continue to advance our knowledge of tank bromeliads' multifunctionality responses in a changing climate. Such knowledge is needed to evaluate the potential consequences of these changes for global cycling in the Neotropics.

## Author Contributions

**Thibaut Rota:** writing – original draft, visualization, writing – review and editing, formal analysis, validation, investigation. **Vincent E. J. Jassey:** conceptualization, investigation, writing – original draft, methodology, validation, writing – review and editing. **Céline Leroy:** investigation, conceptualization, methodology, validation, writing – review and editing. **Jean‐François Carrias:** investigation, methodology, validation, writing – review and editing, conceptualization. **Bruno Corbara:** investigation, methodology, validation, writing – review and editing, conceptualization. **Joséphine Leflaive:** investigation, methodology, validation, writing – review and editing, conceptualization. **Arthur Compin:** investigation, writing – review and editing. **Diane S. Srivastava:** writing – review and editing. **Vinicius F. Farjalla:** writing – review and editing, conceptualization, methodology. **Régis Céréghino:** conceptualization, investigation, funding acquisition, writing – original draft, methodology, validation, writing – review and editing, data curation, supervision, resources, project administration.

## Conflicts of Interest

The authors declare no conflicts of interest.

## Supporting information


**Data S1:** gcb70777‐sup‐0001‐Supinfo.docx.

## Data Availability

The data and R code used for the statistical analyses and graphics are publicly and permanently available on Figshare (CC BY 4.0): https://doi.org/10.6084/m9.figshare.29485832 (Rota 2026, Figshare, DOI: 10.6084/m9.figshare.29485832).
