# Supplementary material for: Extreme Droughts Push Heterotrophic Functions Above Baseline Levels in a Neotropical Ecosystem
Source: Glob Chang Biol. 2026 Mar 3;32(3):e70777. doi: 10.1111/gcb.70777 (PMC12954564; doi:10.1111/gcb.70777)
Supplement: Supplementary file 1 — Data S1: gcb70777‐sup‐0001‐Supinfo.docx. [file GCB-32-e70777-s001.docx]

Supplementary Materials

**Extreme droughts push heterotrophic functions above baseline levels in a Neotropical ecosystem**

Running title: Multifunctional resilience to drought

Thibaut Rota, Vincent E. J. Jassey, Céline Leroy, Jean-François Carrias, Bruno Corbara, Joséphine Leflaive, Arthur Compin, Diane S. Srivastava, Vinicius F. Farjalla and Régis Céréghino


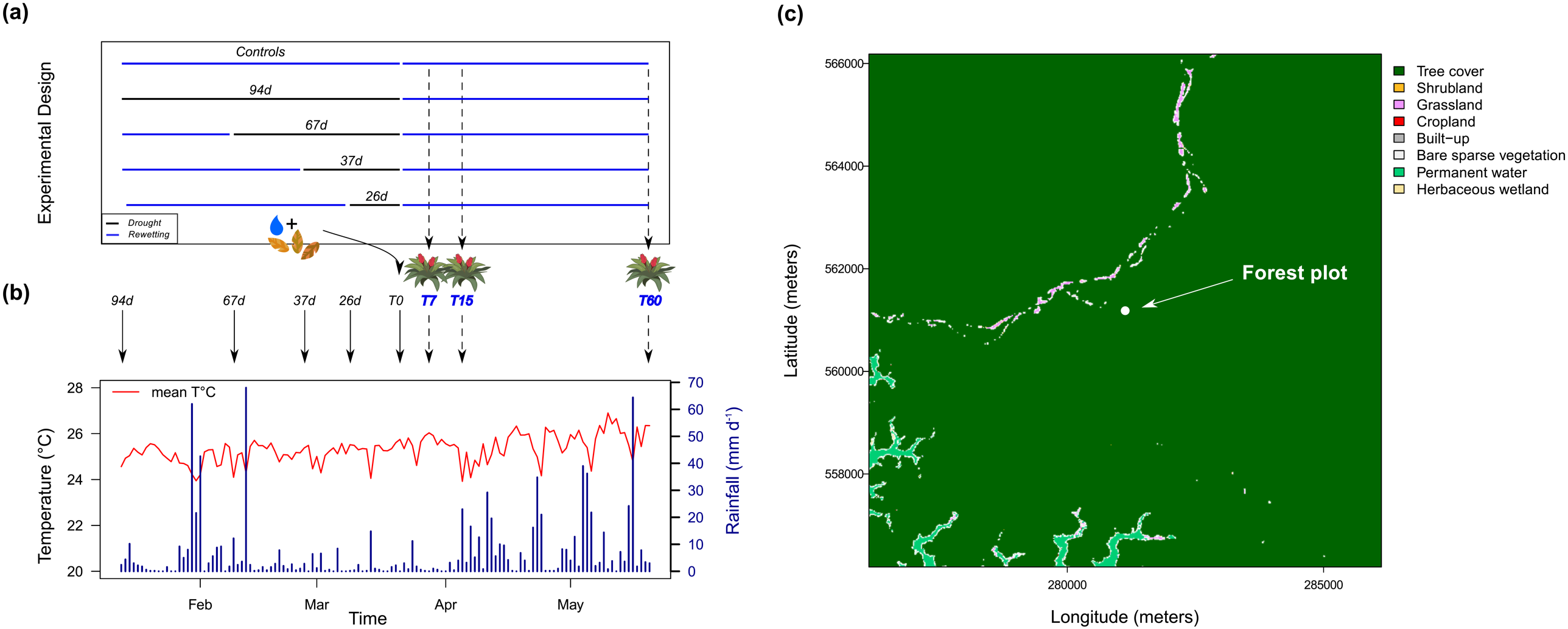


**Fig. S1**. (a) Temporal design of the experiment (dry phase in black, control tank bromeliads and rewetting phase in blue, T_0_ with addition of rainwater and leaf-litter, and T_7_, T_15_ and T_60_ sampling dates). (b) Schedule of drought start dates (black numbers with arrows) and rewetting end dates (blue numbers with arrows) over five months of the experiment, in relation to concurrent patterns in daily mean temperatures (in red, in °C) and daily rainfall time series (blue barplots, in mm) as recorded by the Paracou weather station, 8km from the study site. (c) Map of land cover (European Spatial Agency ‘ESA’ land cover, 2021) for the studied primary rainforest location (dark green), showing the pristine nature of the forest plot studied (white dot), and surrounding area near Petit Saut Reservoir, in French Guyana (impounded lake caused by a dam, shown in turquoise). Longitude and latitudes are in meters, projection “EPSG:32622”. [Map lines delineate study areas and do not necessarily depict accepted national boundaries].

**Table S1**. List of macroinvertebrate taxa recovered from the 135 experimental tank bromeliads of the study, with their per capita dry mass (mg), and corresponding functional feeding group (FFG). We excluded the ‘Piercer’ FFG from our analyses as they occurred in only three tank bromeliads and represented only 0.01% of the FFG biomass.

| Taxa | Per Capita Dry Mass (mg) | Functional Feeding Group (FFG) |
| --- | --- | --- |
| Elpidium bromeliarum | 0.0172 | Deposit feeder |
| Cyphon sp. | 0.2685 | Scraper |
| Scirtes sp. | 0.072 | Scraper |
| Aulophorus.superterrenus | 0.056 | Deposit feeder |
| Corethrella | 0.0559 | Predator |
| Wyeomyia aphobema | 0.0917 | Filter feeder |
| Anopheles nevai | 0.024 | Filter feeder |
| Culex sp1 | 0.1044 | Filter feeder |
| Microculex stonei | 0.0157 | Filter feeder |
| Bezzia sp2 | 0.0511 | Predator |
| Trentepohlia sp1 | 0.5882 | Shredder |
| Chironomini | 0.01846 | Deposit feeder |
| Coenagrionidae | 0.0675 | Predator |
| Orthocladinae | 0.08 | Scraper |
| Trentepohlia sp2 | 0.16 | Shredder |
| Copelatus.larva | 0.254 | Predator |
| Sphaeridinae | 0.0672 | Predator |
| Elmidae | 0.035 | Scraper |
| Paravelia | 1.065 | Predator |
| Cecidomyiidae | 0.02 | Piercer |
| Acari | 0.003 | Deposit feeder |
| Enchytraeidae | 0.195 | Deposit feeder |
| Ostracoda sp2 | 0.007 | Deposit feeder |
| Turbellaria | 0.0003 | Predator |


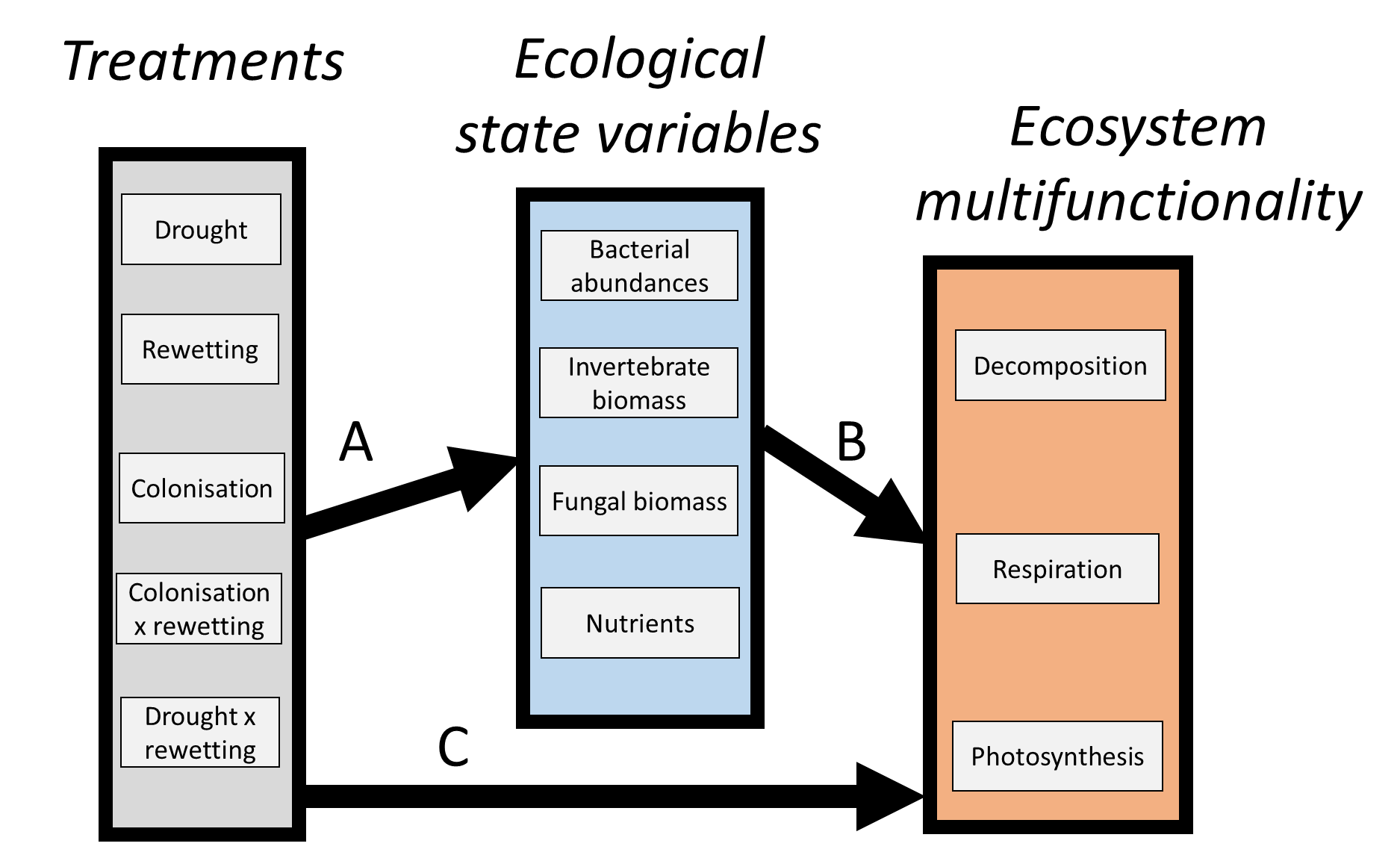


**Fig. S2**. Acyclic graph diagram of the meta structural equation model (meta-SEM) showing the rationale of the general SEM approach we employed to interrogate how abiotic and biotic ecological state variables could mediate the effects of drought and the recovery of ecosystem multifunctionality in tank bromeliads (through indirect paths ***A*** 🡪 ***B***), versus the direct effect of treatments (paths ***C***) or ecological state variables (paths ***B***).


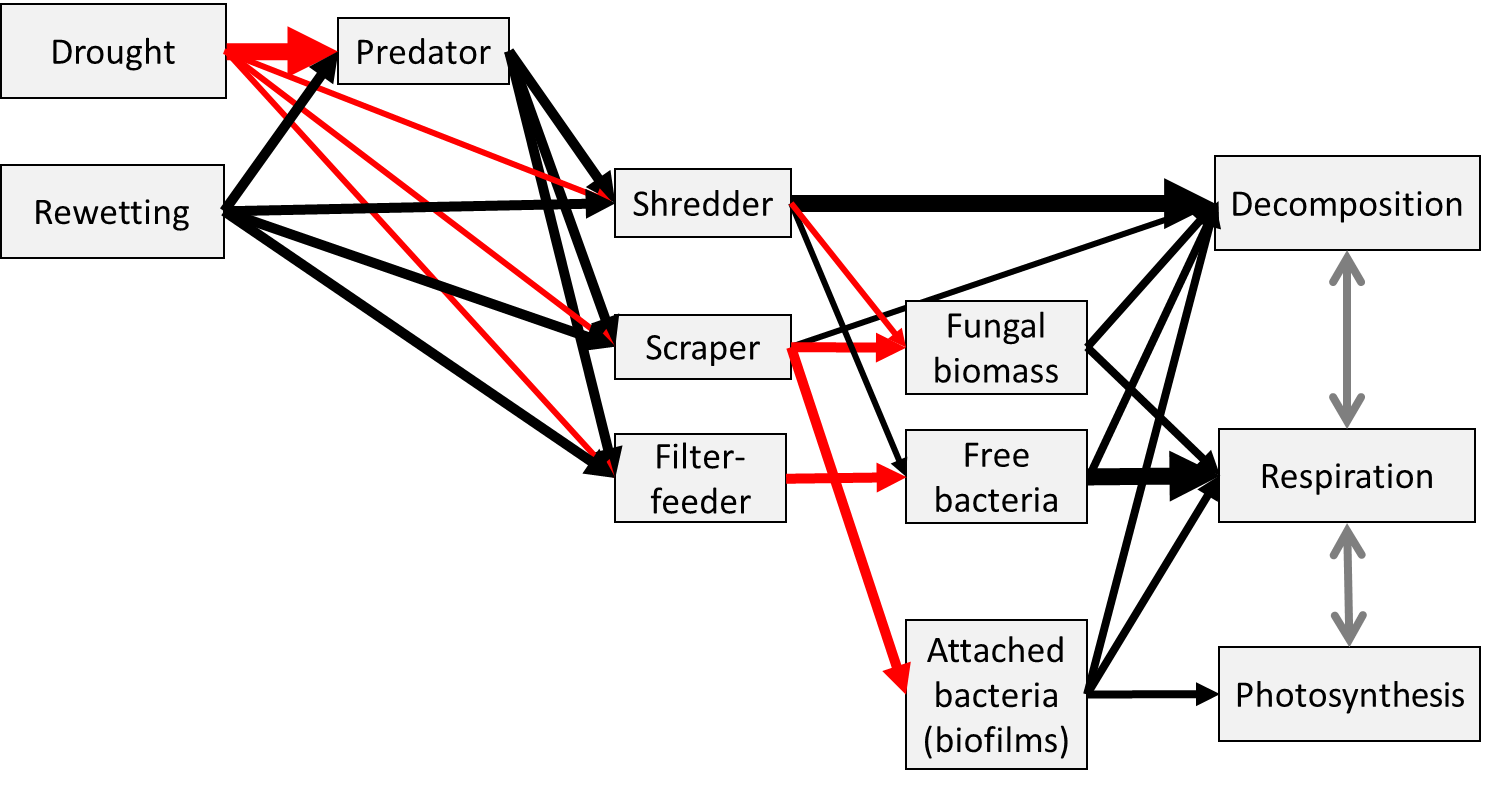


**Fig. S3**. Expended view of the acyclic graph diagram of the meta structural equation model (meta-SEM) (general view in Fig. S2), used to test (i) the effects of drought and rewetting on functional feeding groups (FFGs) of macroinvertebrates (predators, shredders, scrapers, and filter-feeders), and (ii) how these groups could relate to the different microorganism compartments we measured (fungal biomass, free bacteria and attached bacteria), and (iii) the ecosystem functions underlying multifunctionality in our system. Please note that as we had no a priori expectation on the role of deposit-feeders for the functions studied, we did not included that FFG in SEM analyses. Black arrows show paths a priori expected to be positive, and red arrows show paths a priori expected to be negative. Double grey arrows show paths for which directionality and sign would not be easily predicted a priori. The size of arrows is proportional to the magnitude of the a priori effect, e.g., the negative effect of drought was a priori expected to be stronger on predator biomass than that of secondary consumers.

**Table S2**. Summary of statistics of the retained structural equation model (SEM) presented in the main text, after backward variable selection of non-significant paths from a full model. Beta coefficients are standardized coefficients.

| Response | Predictor | β | SE | Df | Crit.Value | P |  |
| --- | --- | --- | --- | --- | --- | --- | --- |
| Shredder biomass | Rewetting × recolonization | 0.3475 | 0.0608 | 98 | 3.6691 | 0.000 | *** |
| P_tot_ | DOC | 0.4485 | 0.0832 | 96 | 5.5122 | 0.000 | *** |
| P_tot_ | Rewetting | 0.3201 | 0.0803 | 96 | 3.9337 | 0.000 | *** |
| P_tot_ | Recolonization | –0.2211 | 0.1639 | 96 | –2.7169 | 0.008 | ** |
| N_tot_ | DOC | 0.3391 | 0.0913 | 98 | 3.5685 | 0.001 | *** |
| Free bacterial densities | P_tot_ | 0.2302 | 0.0921 | 98 | 2.3420 | 0.021 | * |
| Attached bacterial densities | P_tot_ | 0.2694 | 0.0857 | 97 | 3.0933 | 0.003 | ** |
| Attached bacterial densities | Rewetting | –0.6224 | 0.0846 | 97 | –7.1457 | 0.000 | *** |
| Litter decomposition | Shredder biomass | 0.2290 | 0.0938 | 95 | 2.4606 | 0.016 | * |
| Litter decomposition | DOC | –0.2069 | 0.1063 | 95 | –2.0908 | 0.039 | * |
| Litter decomposition | P_tot_ | 0.2951 | 0.1083 | 95 | 2.8621 | 0.005 | ** |
| Litter decomposition | Rewetting × recolonization | 0.2788 | 0.0631 | 95 | 2.8577 | 0.005 | ** |
| Phytosynthesis efficiency | Attached bacterial densities | 0.2577 | 0.0836 | 96 | 3.1113 | 0.003 | ** |
| Phytosynthesis efficiency | Drought | 0.5332 | 0.0836 | 96 | 6.3857 | 0.000 | *** |
| Phytosynthesis efficiency | Drought × Rewetting | –0.2062 | 0.0838 | 96 | –2.4626 | 0.016 | * |
| Microbial CO_2_ repiration | Shredder biomass | 0.2069 | 0.0605 | 96 | 2.9455 | 0.004 | ** |
| Microbial CO_2_ repiration | Free bacterial densities | 0.4631 | 0.0677 | 96 | 6.5531 | 0.000 | *** |
| Microbial CO_2_ repiration | N_tot_ | 0.4790 | 0.0676 | 96 | 6.7749 | 0.000 | *** |

**Fig. S4**. Post drought dynamics of four biological groups: (a) shredder biomass, (b) free bacteria density, (c) attached bacteria density, and (d) fungal biomass (proxied by ergosterol leaf content). In each case, values are expressed as percent deviation from control bromeliads. Dashed and solid regression lines are non-significant and significant regressions, respectively. Coloured bandwidths show 95% confidence intervals. Blue lines and grey bandwidths indicate the treatment with a net to prevent colonisation (only plotted for cases where a rewetting × recolonization interaction was significant).


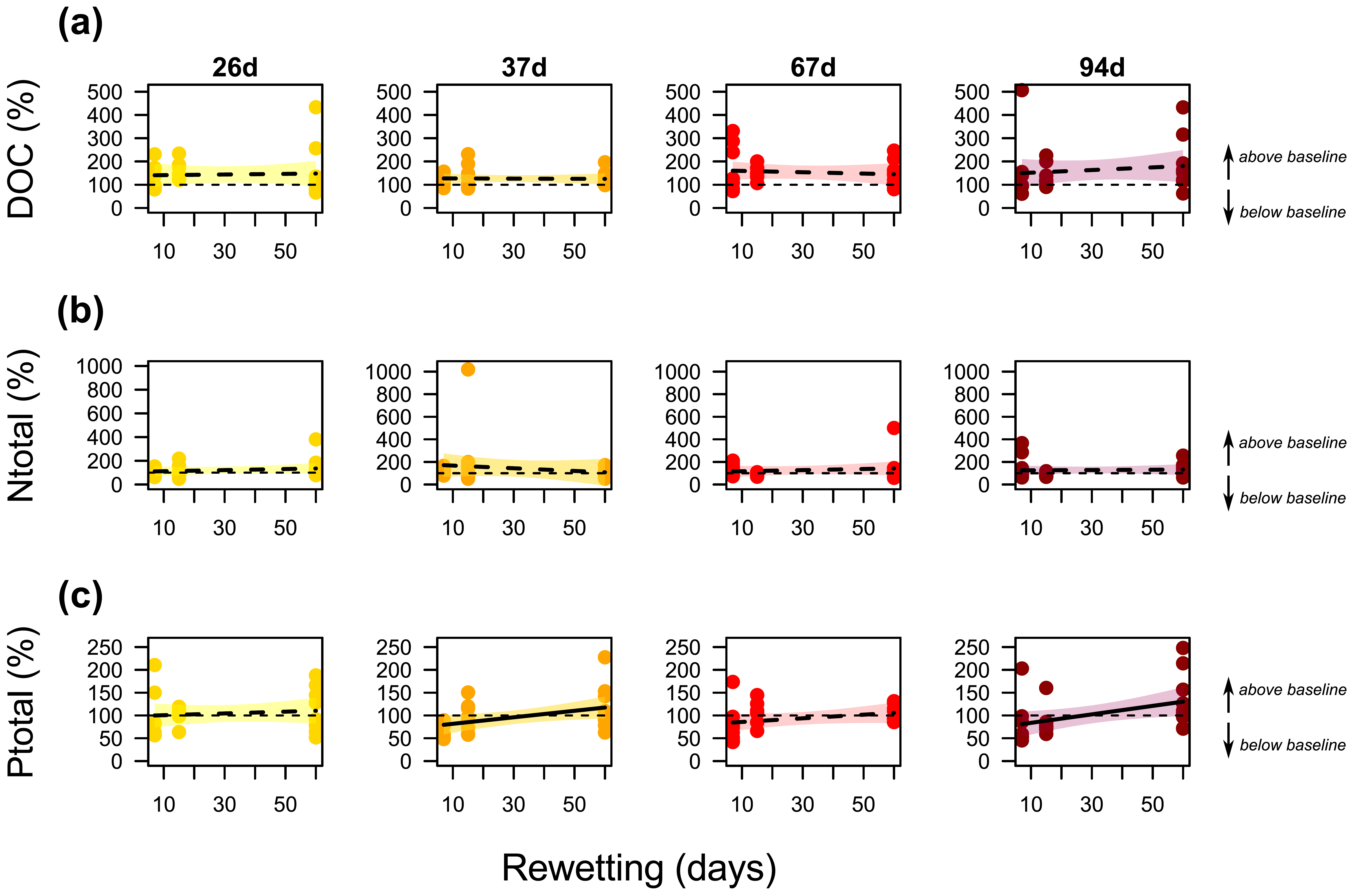


**Fig. S5**. Post drought dynamics of three nutrients found to affect ecosystem functions in this study: (a) DOC, (b) Ntotal, and (c) Ptotal. In each case, values are expressed as percent deviation from control bromeliads. Dashed and solid regression lines are non-significant and significant regressions, respectively. Coloured bandwidths show 95% confidence intervals.

**Fig. S6**. Boxplots showing the relationship between realized dry bromeliad days (i.e., number of days a bromeliad was found entirely dry, y-axis) and the drought treatments (number of days bromeliads have been covered by transparent tarpaulins, x-axis). Statistics are given in the main text.


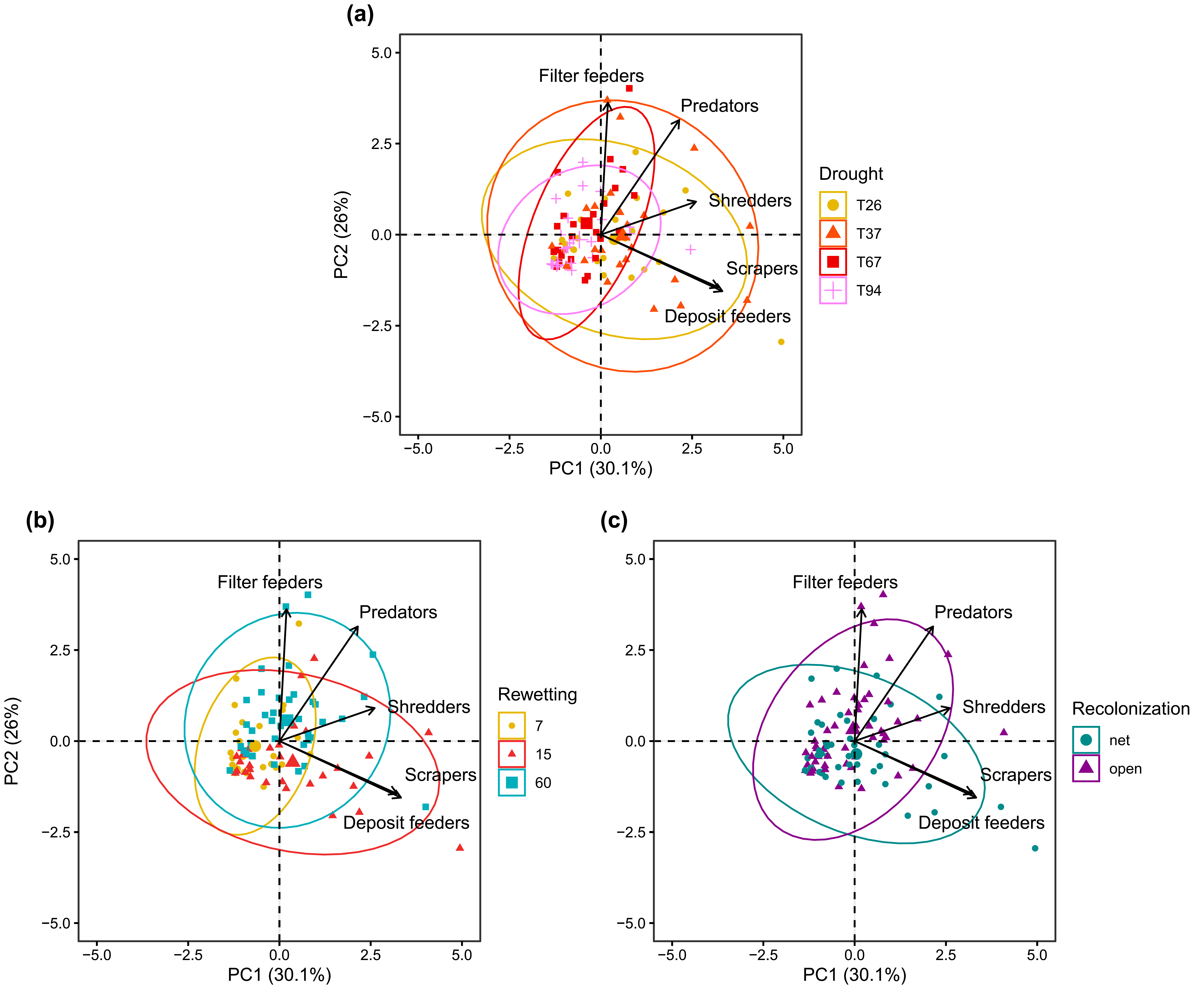


**Fig. S7**. Principal Component Analysis (PCA) biplots of the first two principal components of the dynamics (expressed as percent deviation from baseline) of macroinvertebrate functional feeding groups (FFGs) biomass (a) as a function of drought intensification, (b) at different times during the rewetting period, and (c) between netted (no external recolonization allowed) and open tank bromeliads (external recolonization allowed).

**Table S3**. Permutational Multivariate Analysis of Variance (PERMANOVA) statistics of the effects of treatments on the multivariate space of macroinvertebrate functional feeding groups (FFGs), in percent deviation from controls (and *z-*standardized).

| Variable | Df | SS | *F* | *R*² | *P* |
| --- | --- | --- | --- | --- | --- |
| Drought | 3 | 33.47 | 2.48 | 0.07 | 0.003 |
| Rewetting  Recolonization (net vs open) | 1  1 | 30.00  13.44 | 6.66  2.98 | 0.06  0.03 | 0.001  0.009 |
| Drought × rewetting | 3 | 6.93 | 0.51 | 0.01 | 0.924 |
| Recolonization × rewetting | 1 | 5.66 | 1.26 | 0.01 | 0.293 |
| Residuals | 90 | 405.50 |  | 0.82 |  |


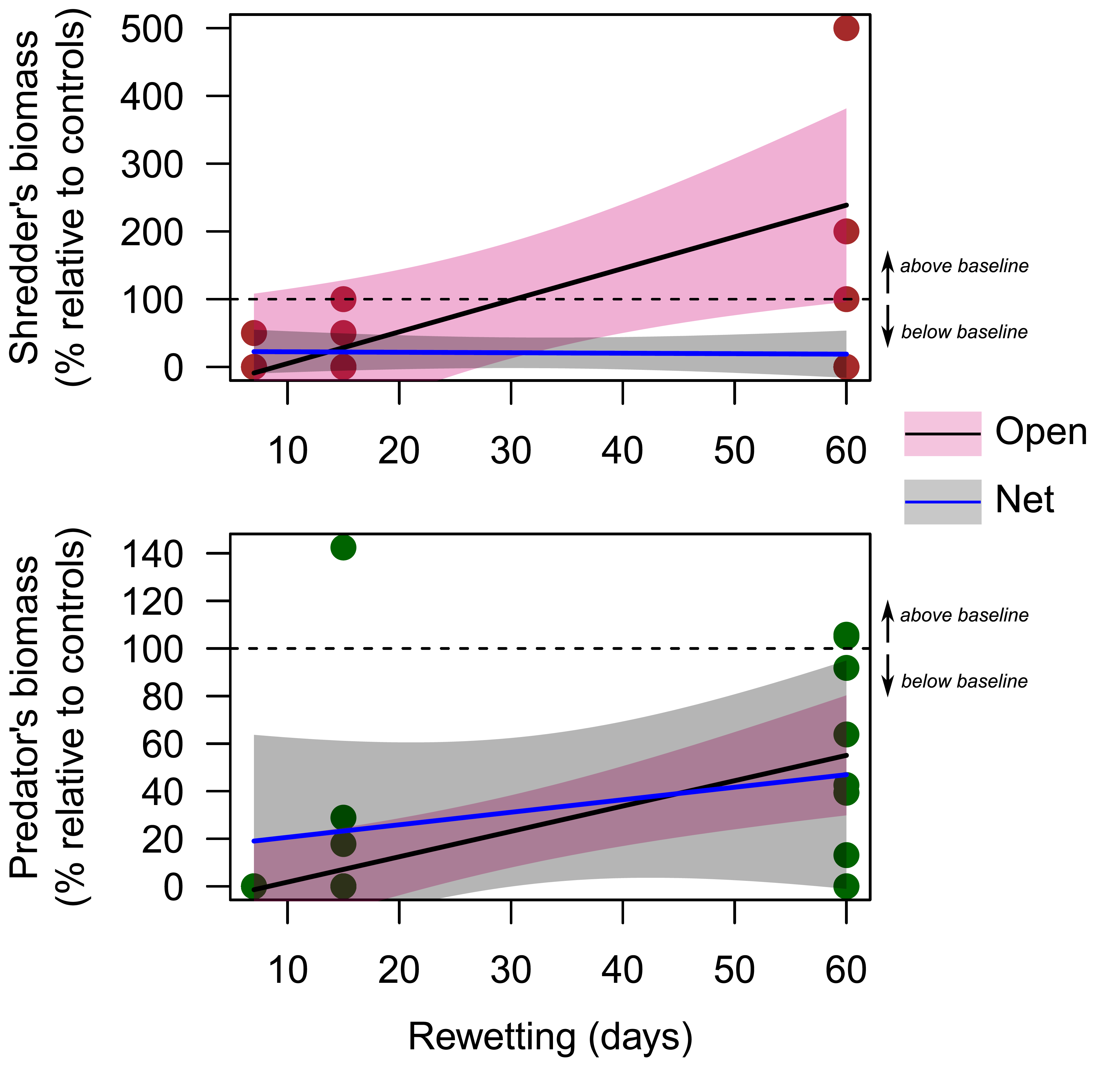


**Fig. S8**. Post drought dynamics of shredder biomass (a) and predator biomass (b) expressed as percentage deviation from baseline conditions (control tank bromeliads), following a 94 days drought (the harshest drought simulated in our experiment). Tank bromeliads that remained open to macroinvertebrate colonization during the rewetting stage are shown with a black regression line with confidence intervals indicated by purple bandwidths (95% CIs); netted tank bromeliads are shown with a blue regression line and grey 95% CIs bandwidths. We observed a significant rewetting-by-recolonization interaction for shredder biomass, but not for predator biomass. Both shredders and predators were significantly affected by the 94d drought scenario, with their biomass reduced by virtually 100% at 7 days of rewetting following the drought. During rewetting, shredder biomass increased by 4.7% by day in open bromeliad conditions, to finish 2-times above baseline conditions at 60d rewetting. Predator biomass increased by 1.07% by day (i.e., 4-times less than for shredders), and did not recover until to baseline conditions at 60 days of rewetting.

**Appendix 2**. *Upscaling observations of C-CO_2_ emissions from tank bromeliads following droughts to the Neotropics*

**Summary of the approach**

Based on basic knowledge of post drought microbial dynamics, we aimed at upscaling the net increase in microbial C-respiration following drought events that we observed to a conservative estimate of water volume held by bromeliads in the Neotropics, for a year, and relative to control, undisturbed bromeliads. In brief, we used a piecewise model combining our linear observations followed by an exponential decay model of bacterial C-respiration (the increase in microbial activity following drought events is likely to show a spike followed by an abrupt exponential decrease, with varying and unknown slope given by the exponential decay exponent ‘*K’*; (Brangarí et al., 2020). The piecewise model we built by combining these two functions is very similar to the post-drought dynamics of microbial respiration in most known systems, such as soils (Brangarí et al., 2020). We then parametrized and ran simulations for varying decay exponent ‘*K*’ (0.001, 0.1, and 0.1) by using our observed measurements and propagated uncertainties from Monte Carlo Markov Chain (MCMC) posterior distributions for each discrete hour over a year. We then obtained mean total emitted C-CO_2_ mL^-1^ for a year with the sum of hourly rates over the year, and upscaled these quantities to the surface of Amazonian rainforests (i.e., 5.94 10^6^ km^2^; Papastefanou et al., 2022), hosting on average 3,558 bromeliads ha^-1^ as a conservative estimate (Dézerald et al., 2018), with an average volume of 115 ml as measured in our experiment. Since droughts are not expected to affect the entirety of the Amazonian basin, we simulated yearly emitted C-CO_2_ for droughts covering either 5%, 10% or 50% of the Amazonian basin surface area, following observations made by Papastefanou et al. (2022). All these parameters are conservative regarding the literature (Martinson et al., 2010; Goffredi, Jang, et al., 2011; Lehours et al., 2016).

**Methods**

We defined the following function$f(T,m,K,x0,y0,yf)$,

with $T$ representing the rewetting time following the end of the drought (*T=60 days*), $m$ the slopes of our linear observations between C-CO_2_ emission rate and time *T*, $K$ the decay exponent shaping the steepness of the return to the baseline, $x0$ the piecewise breaking point (here, our maximum rewetting time of 60 days), $y0$ the C-CO_2_ emission rate value at the piecewise breaking point, and $yf$ the baseline C-CO_2_ emission rate value in control, undisturbed bromeliads. The piecewise model was written as follows:

If $T \leq x0;$

$y\left( T \right)=m T+y0-m x0$ (1a)

If $T\geq x0$;

$y\left( T \right)=yf+\left( y0-yf \right) e(-K\left( T-x0 \right))$ (1b)

We then performed 100 simulations of C-CO_2_ emission rates across discrete $T$ values from $Tij \left\{ 0, 6504 \right\} (h)$ compared to controls, for each of the 1,000 values sampled from parameters in $m,K,x0,y0$ and $yf$, with:

$m \sim N\left( slope, \sigma^{2} \right)$ of MCMC linear estimate based on our observations;

$K= 0.001;0.01 or 0.1$.

$x0= 1440 hours (fixed to 60days)$,

$y0 \sim N(Intercept at 1440h, \sigma^{2})$ of MCMC linear estimate based on our observations,

$yf=0$ (since we re-ran the linear regressions as relative to controls).

We recorded the average and 95% confidence intervals of every simulation of C-CO2 emission rates (µg C-CO_2_ mL^-1^ h^-1^) for every hour of a year.

Then, we used the following equation to extrapolate C-CO_2_ emission rates and 95% CI (expressed relative to the norm) to the total C-CO_2_ emitted over a year and for all bromeliads in the Neotropics, following the different drought scenarios. Please note that we assume here that emission rates during the 94d of drought are equal to controls.

To perform this extrapolation, we summed the fluxes of C-CO_2_ $y(x)ij$ that we had simulated for any discrete hour over a year (here we assumed no microbial respiration during the drought), for each time step *T*$ij \left\{ 0, 6504 \right\} (in hour)$, therefore assuming that each bromeliad experienced the maximum number of days drought (94 days). This time-discrete integration was written as follows:

$C-CO2 emission (\mu g C-CO2\mathrm{mL}-1 yr-1) = \frac{\sum y(x)ij}{\sum Tij(h)}$ (2)

As a conservative estimate, Neotropical forests (i.e., 6 × 10^6^ km^2^) could exhibit 3’558 bromeliads ha^-1^ at our study location (Dézerald et al., 2018), for an average volume of 115 ml (our experiment), which corresponds to conservative values regarding the other informations found in the literature (Goffredi, Kantor, et al., 2011; Lehours et al., 2016). We finally converted the obtained C-CO2 emissions for all bromeliads of the Neotropical forests (µgC-CO2 yr^-1^) into Petagrams (Pg C-CO2 yr^-1^).

**Results of the simulation**

Our sensitivity analysis of the parameter $K$ (decay rate of microbial C-respiration) only affected our predictions to a small extent (Fig. S9a). Unsurprisingly, the strongest effect on our estimates came from different scenarios for percent cover of drought events over the Amazonian basin (Figure S9b). Averaging across *K* parameters, the mean increase in microbial C-respiration following drought events of varying intensities could represent an increase in annual emission rates of 0.2 Pg C-CO_2_ (95% confidence intervals: 0.1 – 0.3) for 5% of drought coverage, 0.4 Pg C-CO_2_ (0.2 – 0.6) 95% CI for 10% of drought coverage, and 2 Pg C-CO_2_ (1.1 – 2.9) 95% CI for 50% of drought coverage (Fig. S9b).

**Fig. S9**. (a) Simulations of estimated C-CO2 emission rates over a year following our drought scenarios. We show 95% confidence interval bandwidths only for the treatment of 94d of drought (light pink bandwidth), since it was the only treatment showing a significant increase in microbial CO_2_ respiration following drought. (b) Based on these simulations of rates, we estimated additional yearly C-CO_2_ emissions (in Petagram; Pg C yr^-1^) relative to baseline levels from drying tank bromeliads following drought intensities ranging from 26d to 94d, and for three scenarios of Amazonian basin’s drought percentage covers (5%, 10%, 50%; following Papastefanou et al., 2022), and decaying exponent *K* (0.001, 0.01, and 0.1).

**References**

Brangarí, A. C., Manzoni, S., & Rousk, J. (2020). A soil microbial model to analyze decoupled microbial growth and respiration during soil drying and rewetting. *Soil Biology and Biochemistry*, *148*, 107871. https://doi.org/10.1016/j.soilbio.2020.107871

Dézerald, O., Leroy, C., Corbara, B., Dejean, A., Talaga, S., & Céréghino, R. (2018). Tank bromeliads sustain high secondary production in neotropical forests. *Aquatic Sciences*, *80*(2). https://doi.org/10.1007/s00027-018-0566-3

Goffredi, S. K., Jang, G. E., Woodside, W. T., & Ussler, W. (2011). Bromeliad Catchments as Habitats for Methanogenesis in Tropical Rainforest Canopies. *Frontiers in Microbiology*, *2*. https://doi.org/10.3389/fmicb.2011.00256

Goffredi, S. K., Kantor, A. H., & Woodside, W. T. (2011). Aquatic Microbial Habitats Within a Neotropical Rainforest: Bromeliads and pH-Associated Trends in Bacterial Diversity and Composition. *Microbial Ecology*, *61*(3), 529–542. https://doi.org/10.1007/s00248-010-9781-8

Lehours, A.-C., Jeune, A.-H. L., Aguer, J.-P., Céréghino, R., Corbara, B., Kéraval, B., Leroy, C., Perrière, F., Jeanthon, C., & Carrias, J.-F. (2016). Unexpectedly high bacteriochlorophyll *a* concentrations in neotropical tank bromeliads: Anoxygenic phototrophy in tank bromeliads. *Environmental Microbiology Reports*, *8*(5), 689–698. https://doi.org/10.1111/1758-2229.12426

Martinson, G. O., Werner, F. A., Scherber, C., Conrad, R., Corre, M. D., Flessa, H., Wolf, K., Klose, M., Gradstein, S. R., & Veldkamp, E. (2010). Methane emissions from tank bromeliads in neotropical forests. *Nature Geoscience*, *3*(11), 766–769. https://doi.org/10.1038/ngeo980

Papastefanou, P., Zang, C. S., Angelov, Z., de Castro, A. A., Jimenez, J. C., De Rezende, L. F. C., Ruscica, R. C., Sakschewski, B., Sörensson, A. A., Thonicke, K., Vera, C., Viovy, N., Von Randow, C., & Rammig, A. (2022). Recent extreme drought events in the Amazon rainforest: Assessment of different precipitation and evapotranspiration datasets and drought indicators. *Biogeosciences*, *19*(16), 3843–3861. https://doi.org/10.5194/bg-19-3843-2022
